# Automatic Adaptation to Fast Input Changes in a Time-Invariant Neural Circuit

**DOI:** 10.1371/journal.pcbi.1004315

**Published:** 2015-08-06

**Authors:** Arjun Bharioke, Dmitri B. Chklovskii

**Affiliations:** Janelia Research Campus, Howard Hughes Medical Institute, Ashburn, Virginia, United States of America; Northwestern University, UNITED STATES

## Abstract

Neurons must faithfully encode signals that can vary over many orders of magnitude despite having only limited dynamic ranges. For a correlated signal, this dynamic range constraint can be relieved by subtracting away components of the signal that can be predicted from the past, a strategy known as predictive coding, that relies on learning the input statistics. However, the statistics of input natural signals can also vary over very short time scales e.g., following saccades across a visual scene. To maintain a reduced transmission cost to signals with rapidly varying statistics, neuronal circuits implementing predictive coding must also rapidly adapt their properties. Experimentally, in different sensory modalities, sensory neurons have shown such adaptations within 100 ms of an input change. Here, we show first that linear neurons connected in a feedback inhibitory circuit can implement predictive coding. We then show that adding a rectification nonlinearity to such a feedback inhibitory circuit allows it to automatically adapt and approximate the performance of an optimal linear predictive coding network, over a wide range of inputs, while keeping its underlying temporal and synaptic properties unchanged. We demonstrate that the resulting changes to the linearized temporal filters of this nonlinear network match the fast adaptations observed experimentally in different sensory modalities, in different vertebrate species. Therefore, the nonlinear feedback inhibitory network can provide automatic adaptation to fast varying signals, maintaining the dynamic range necessary for accurate neuronal transmission of natural inputs.

## Author Summary

An animal exploring a natural scene receives sensory inputs that vary, rapidly, over many orders of magnitude. Neurons must transmit these inputs faithfully despite both their limited dynamic range and relatively slow adaptation time scales. One well-accepted strategy for transmitting signals through limited dynamic range channels–predictive coding–transmits only components of the signal that cannot be predicted from the past. Predictive coding algorithms respond maximally to unexpected inputs, making them appealing in describing sensory transmission. However, recent experimental evidence has shown that neuronal circuits adapt quickly, to respond optimally following rapid input changes. Here, we reconcile the predictive coding algorithm with this automatic adaptation, by introducing a fixed nonlinearity into a predictive coding circuit. The resulting network automatically “adapts” its linearized response to different inputs. Indeed, it approximates the performance of an optimal linear circuit implementing predictive coding, without having to vary its internal parameters. Further, adding this nonlinearity to the predictive coding circuit still allows the input to be compressed losslessly, allowing for additional downstream manipulations. Finally, we demonstrate that the nonlinear circuit dynamics match responses in both auditory and visual neurons. Therefore, we believe that this nonlinear circuit may be a general circuit motif that can be applied in different neural circuits, whenever it is necessary to provide an automatic improvement in the quality of the transmitted signal, for a fast varying input distribution.

## Introduction

Early sensory processing faces the challenge of communicating sensory inputs with large dynamic range to the rest of the brain using neurons with limited dynamic range [1–6]. In electrical engineering, such challenges are met by compressing the inputs using predictive coding [7–11]. Given this, Srinivasan et al. conjectured that the early visual systems of both vertebrates and invertebrates may also implement predictive coding [12]. More recently, predictive coding has also been conjectured to function in cortical visual and auditory processing, and attention [13–16].

**Fig 1:**
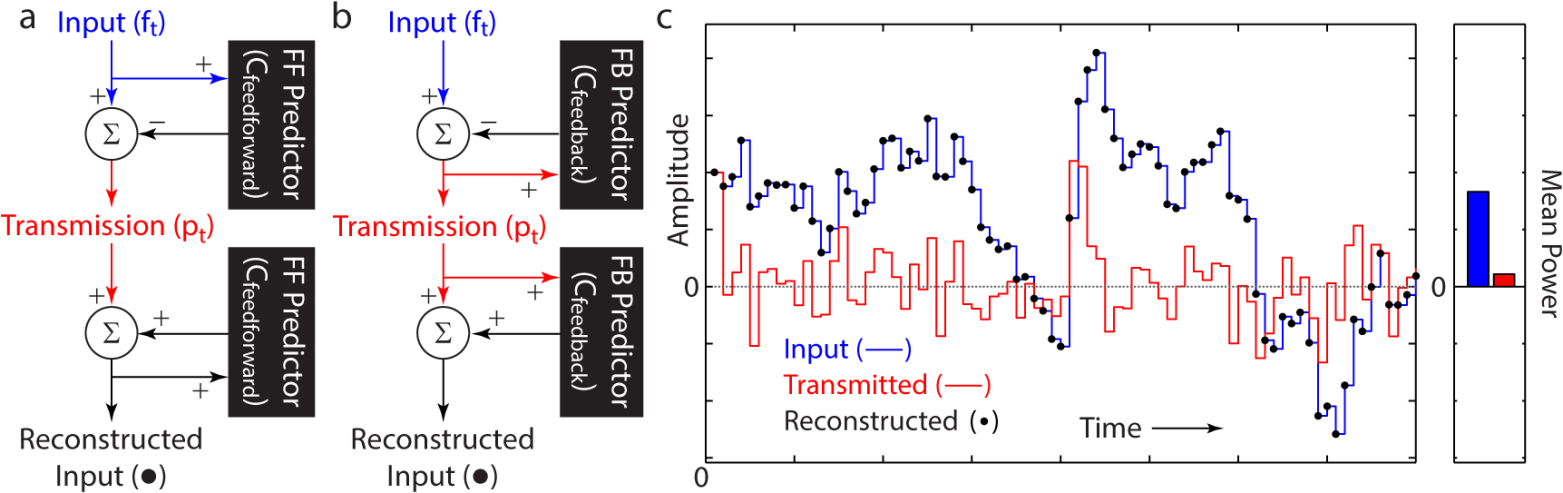
Lossless compression via predictive coding. Feedforward (a) and feedback (b) predictive coding circuits (as in Eq (1)), showing the circuit structure necessary for reconstructing the input. (c) Lossless reconstruction (black dots) of an input (blue) from the transmitted output of a predictive coding circuit (red). The mean power up to each point in time (solid bars) for the input (blue) and transmission (red) demonstrates the reduction in the mean transmitted power via predictive coding.

A predictive coding circuit attempts to reduce the dynamic range of an input by subtracting a prediction of the current input value–based on past input values–from the actual current input value, and then transmitting only the difference, i.e. the prediction error (Fig 1). Such a strategy only works if the prediction is good, which requires the existence of stationary (predictable) correlations within the input, and the ability of the algorithm to adapt to them. For example, the optimal linear prediction-error filter, that minimizes the relative power of the transmission, depends on the signal-to-noise ratio (SNR) of the inputs [7–9]. Indeed, in the invertebrate visual system, Srinivasan et al. observed adaptations to the prediction-error filter in response to changes in the input SNR [12].

This adaptation of the response filters of a neuron to changing input properties has been explored in the literature [2,3,12,17–21]. This includes the predictive coding formulation introduced by Srinivasan et al. [12], which was also extended to the vertebrate visual system [20]. Indeed, Hosoya et al. [20] demonstrated that predictive coding can explain changes to the response filters of retinal ganglion cells, for adaptations on the time scale of seconds. Other authors [22–24] alternatively postulated that sensory systems must whiten input signals. They showed that varying response filters to whiten the input signals, for different inputs, also matches the observed changes in neurons’ responses.

However, the adaptation of early sensory processing circuits must also be very fast, since the input statistics often vary rapidly, e.g. across a saccade [2,3,25], or during movements within a complex auditory environment [26]. Many of the adaptations that have been proposed in the literature vary the biophysical properties of neurons; hence, they are thought to occur at a slower time scale than the speed at which input changes occur. Nevertheless, experimental measurements of the linear filters of sensory neurons have shown that they do indeed change rapidly–often as fast as can be experimentally measured [27,28]. Therefore, to maintain optimality, linear response filters in models of sensory coding must adapt to changes in the input signal statistics–including the input SNR–rapidly, following each input change, yet prior to the next change.

How can a linear filter change at a high rate in response to changes in its input statistics? The addition of a time-invariant nonlinearity can allow the construction of a circuit that “instantaneously adapts” its linearized responses to input changes [28,29]. However, it is not clear if such a circuit exists, and how to construct it. Previous work on the effect of static nonlinearities on neurons has focused mostly on the possible computational functions of a spiking nonlinearity on the output of a single neuron [29–32], or a multiplicative nonlinearity within a motion detecting circuit [33].

Here, we demonstrate that a network of leaky integrator neurons, with a threshold nonlinearity, is, indeed, able to achieve automatic adaptation to changes in the ratio of the predictable component of an input to its unpredictable component. Since noise is, by definition, unpredictable, one specific case of this would be sudden changes to the input SNR. We first show that, for certain stimulus ensembles, linear leaky integrator neurons can implement linear predictive coding through both a feedback and a feedforward inhibitory circuit. We find the parameters that allow these networks to minimize the output dynamic range. Comparing these implementations, we find that the structure of the adaptation in the feedback inhibitory circuit lends itself to the construction of an automatically adapting filter. Specifically, the addition of a biologically-plausible threshold nonlinearity to the feedback inhibitory circuit allows it to approximate the performance of the optimal linear filter over a range of input SNRs.

We compare the responses of a nonlinear predictive coding circuit to available experimental results. The instantaneous changes to the linearized filter of the nonlinear circuit match the fast changes measured to the linear filters of neurons in different sensory modalities, in response to rapid input changes. Hence, our results support the nonlinear feedback inhibitory circuit as a circuit implementation of predictive coding that models the response of early processing in various sensory modalities, facilitating the transmission of rapidly varying, high dynamic range inputs, through slow, low dynamic range neurons.

## Results

### Predictive Coding Framework

In the field of adaptive signal processing, predictive coding algorithms have commonly been used for signal compression [7,10,34], including in the transmission of telecommunications signals (e.g. in the GSM standard) [35,36] and in video compression [37–39]. These algorithms compute a prediction of the current input based on previous values of the input, and then subtract it away from the actual current input (Fig 1A and 1B). If the signal possesses some statistical structure, an accurate prediction can be made and the output transmission will have a smaller power than the input (Fig 1C and 1D) [7–11].

In a general predictive coding algorithm, acting on an input time series {*f*_*t*_}, the prediction can be constructed in two ways: (1) from past values of the input signal, or (2) from past values of the transmitted output of the algorithm. In (1), the algorithm is entirely feedforward (Fig 1A). In contrast, in (2), the algorithm is feedback (Fig 1B). In each case, we can write the algorithm as:

$$p_{t}=f_{t}-C_{feedforward}\left(f_{t-1},f_{t-2},\ldots \right)\;\text{or}\;p_{t}=f_{t}-C_{feedback}\left(p_{t-1},p_{t-2},\ldots \right)$$ (1)

where *p*_*t*_ is the transmitted signal and the predictions (computed either linearly or nonlinearly) are *C*(⋅).

A crucial property of predictive coding algorithms is that they transmit information losslessly. Specifically, their function is to transmit all the input that they receive including both signal and noise. This is unlike many other algorithms commonly used in neuroscience, which separate signal from noise. Indeed, as structured in Eq (1) and assuming that there is no communication noise in the transmission process, the output of a predictive coding network can be used to reconstruct the entire input, losslessly (Fig 1) [8,11]. Further, this is independent of the linear or nonlinear way in which the prediction is computed (S1 Text). This property is important in our identification of an optimal predictive coding algorithm.

To formulate this optimization problem, we define:

1. A class of allowable predictive coding algorithms, within which to identify an optimal algorithm
2. An input ensemble over which the algorithm is optimized.
3. An optimization metric to measure the algorithm’s performance

### Optimizing a Linear Predictive Coding Algorithm

We start by defining the class of *linear predictive coding algorithms*–predictive coding algorithms in which the prediction is a linear combination of the past inputs:

$$p_{t}=f_{t}-\sum_{i=1}^{\infty }w_{i}\cdot f_{t-i}$$ (2)

We have written this in the feedforward implementation. However, since the equation is linear, this results in no loss of generality; it can be rewritten recursively to obtain the feedback case.

Eq (2) describes a predictive encoding constructed by a class of linear temporal filters, which vary in the way in which they compute the prediction. Each such temporal filter is defined by its set of parameters, {*w*_*i*_}, causally, for all time steps, *i*, counting backwards through time. We now define the input ensemble, and optimization metric with which we can identify a specific temporal filter (i.e. specific set of parameters {*w*_*i*_}) that is optimal.

### Naturalistic Subset of Inputs

Ideally, one would like to find the optimal filter over the space of natural images. However, given the complexity of this space, we chose to use a subset of such inputs. Natural image amplitudes are distributed, over time, with a power law distribution over temporal frequencies [40]. It is possible to decompose such an input (up to some high frequency roll-off) into a sum of several exponentially correlated components, each with a different time constant [41,42].

Therefore, we chose an input composed of one such exponentially correlated signal (with a single time constant, *τ*_*s*_), combined with uncorrelated noise, combined at a particular SNR, *σ* (Methods):

$$f_{t}=\sqrt{\frac{\sigma }{1+\sigma }}s_{t}+\sqrt{\frac{1}{1+\sigma }}\varepsilon_{t}$$ (3)

This input provides the ensemble over which we can identify an optimal linear predictive coding filter. We believe that this subset of inputs is naturalistic, since it should be possible to combine several input subsets (constructed with different time constants) to generalize back to the space of natural images.

### Assessing Performance of Predictive Coding

The final part of the formulation of the optimization problem is a performance metric against which to optimize the filter. Since the goal of applying predictive coding is to reduce the dynamic range required to transmit a signal, a natural measure of performance would be the degree of reduction in the power of the transmitted signal, relative to the input power. We term this the network gain, defined as:

$$\text{Network Gain}\equiv \frac{\text{Transmitted Power}}{\text{Input Power}}=\frac{\frac{1}{t}\sum_{i=1}^{t}{\left(p_{i}\right)}^{2}}{\frac{1}{t}\sum_{i=1}^{t}{\left(f_{i}\right)}^{2}}$$ (4)

for *p*_*i*_ as defined in Eq (2), and *f*_*i*_ as defined in Eq (3).

Ideally, any metric of performance, for a compression algorithm, would include both the degree of compression, and a measure of the information lost due to the compression, e.g. reconstruction error. However, as introduced above, predictive coding algorithms encode inputs losslessly. Hence, any reconstruction error is necessarily zero (Fig 1C). Therefore, we can measure the performance of different predictive coding algorithms just by using the network gain, and we now solve for the optimal linear temporal filter that minimizes this gain over the input ensemble.

### Optimal Linear Predictive Coding Filter

Finding the linear filter that minimizes the network gain is a specific example of a common optimization in the adaptive signal processing literature [7–9], but the specific derivation that we utilize is detailed in S1 Text.

Briefly, we compute the power of the filter by transforming Eq (2) into the frequency domain, and then compute the output power by applying the transformed transfer function to the autocorrelation function of the input. We then solve for the optimal values over the set of *w*_*i*_ by differentiation. The solution is a function of the two parameters that characterize the input ensemble, the time constant (*τ*_*s*_) and SNR (*σ*):

$$w_{i}^{*}=\frac{\Lambda^{*}}{1-\Lambda^{*}}\cdot {\left(\beta \cdot \left(1-\Lambda^{*}\right)\right)}^{i}$$ (5)

where

β=e−1τsandΛ*(β,σ)=(β2−1)(1+σ)+(β2−1)β2(σ−1)2−(β2−1)(1+σ)22β2 (6)

It is important to note that:

- *β* is dependent only on the correlation time constant of the signal component
- Λ^*^ is dependent both on the signal, and the SNR

**Fig 2:**
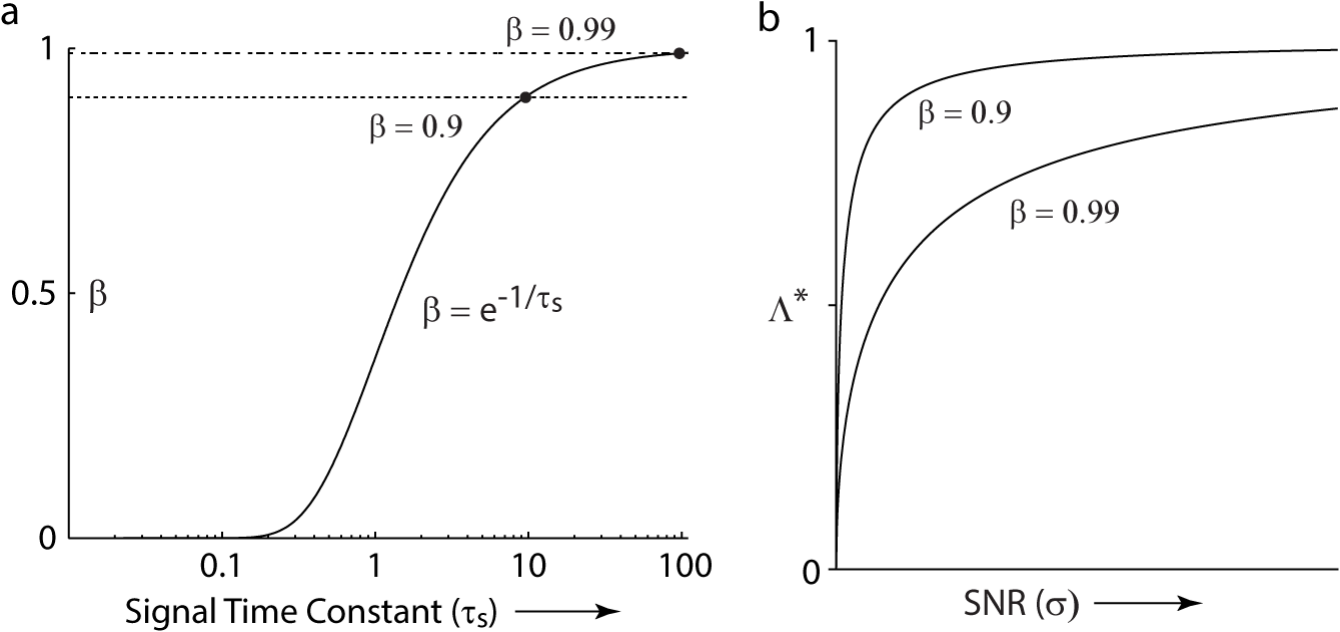
Parameters of the optimized linear predictive coding algorithm. (a) *β* = *β*(*τ*_*s*_) (b) Λ^*^ = Λ^*^(*β*, *σ*): Eq (6) for two values of *β*.

Plotting each of these variables, for some values of the input parameters, provides their qualitative structure. First, *β* varies from 0 to 1, as the correlation time constant of the signal increases (Fig 2A). Second, for each fixed *β*, Λ^*^ varies from 0 to 1, as the SNR increases (Fig 2B). This quantitative value of Λ^*^ varies with *β*, but the increase from 0 to 1 always holds, qualitatively.

Substituting Eq (5) back into Eq (2), we can write down the linear predictive coding filter that minimizes the power of the transmitted signal:

$$p_{t}=f_{t}-\frac{\Lambda^{*}}{1-\Lambda^{*}}\sum_{i=1}^{\infty }{\left(\beta \cdot \left(1-\Lambda^{*}\right)\right)}^{i}\cdot f_{t-i}$$ (7)

An interesting property of the optimal linear predictive coding algorithm is its structure in the high noise, low signal regime (i.e. low SNR in Fig 2B). In that regime, Λ^*^ → 0. Hence, the prediction approaches 0, and *p*_*t*_ → *f*_*t*_. Initially, this might seem counterintuitive, since the predictive coding network is transmitting the entire, noisy input to downstream circuits. However, any prediction of an uncorrelated input will, on average, increase the power of the transmitted output. Therefore, sending out no prediction does indeed minimize the network gain.

Further intuition about the values of the parameters is most useful when applied to specific implementations of this algorithm. Therefore, we first show that it is possible to implement Eq (7) using a circuit of linear leaky integrator neurons.

### Implementing Optimal Filter with Linear Neurons

A simple model of a biological neuron is a leaky integrator (S1 Fig) [43,44], whose voltage response (v) is modeled by an exponential low pass filter (Methods):

$$v_{t}=\frac{g_{s}}{\tau_{m}}\sum_{i=0}^{\infty }e^{-\frac{i}{\tau_{m}}}v_{t-i}^{input}$$ (8)

where the time constant of the filter, *τ*_*m*_, is the membrane time constant, and g_s_ is the synaptic conductance (measured as a fraction of the membrane conductance).

Comparing Eq (8) to Eq (7), we see that the optimal linear predictive coding filter can be implemented by a combination of two leaky integrator neurons, with different time constants. First, in the limit of *τ*_*m*_ → 0, Eq (8) implies that $v_{t}\propto v_{t}^{input}$. Therefore, the first term in Eq (7) can be implemented by a neuron with a short time constant. Second, the subtracted prediction in Eq (7) is simply an exponential weight on the past inputs and, hence, can also be implemented by a neuron with a specific time constant.

This structure can be implemented with different circuits, and we explore both feedforward and feedback two-neuron circuit implementations of the predictive coding algorithm.

### Feedforward and Feedback Implementations

**Fig 3:**
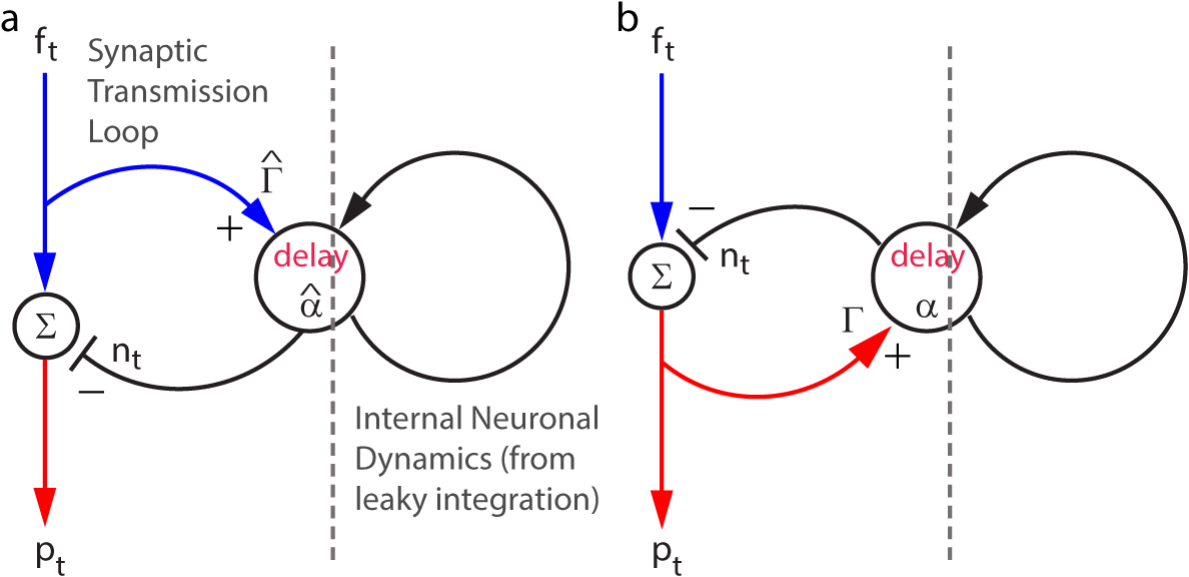
Schematic neural networks implementing predictive coding, through feedforward (a) or feedback (b) inhibition. Networks’ parameters are: (a) Feedforward: $\hat{\alpha }$, $\hat{\Gamma }$. (b) Feedback: *α*, Γ. In both circuits, the unit that outputs *p*_*t*_ is termed the *principal cell*, and the unit that outputs *n*_*t*_ the *interneuron*. There are two synapses onto each interneuron: (1) its external input and (2) a representation of its internal dynamics (right of the dashed line). We can describe its internal dynamics in this way because the interneuron is a leaky integrator, i.e. its internal dynamics (Eq (8)) are an infinite sum of past outputs, discounted at each time step by a fixed multiplier. Therefore, they can be represented by a delayed input across a synapse, with a multiplicative discounting factor, as in (a) and (b).

The feedforward and feedback implementations of predictive coding (Fig 3) are each characterized by two parameters: the time constant of the interneuron (feedforward: $\hat{\alpha }$; feedback: *α*) and the loop gain (feedforward: $\hat{\Gamma }$; feedback: Γ), i.e. the gain of input through the interneuron relative to direct input. For each network, the two parameters may take different values to implement the optimal linear predictive filter (that minimizes network gain for the given input statistics). To derive these values, we first construct a recursive analytical form for each network’s dynamics, following the flow of information around the circuit, and adding a time step delay at the synapses leading into the interneuron (as diagrammed in Fig 3) (Methods).

The recursive dynamics of the feedforward circuit (Fig 3A) are:

$$\{\begin{array}{c} n_{t}=\hat{\alpha }\left(n_{t-1}+\hat{\Gamma }f_{t-1}\right)\\ p_{t}=f_{t}-n_{t} \end{array}$$ (9)

where the interneuron’s time constant is characterized by a discounting factor, $\hat{\alpha }=e^{-1/\tau }<1$, that discounts the voltage from the past time step by a fixed multiple, and the feedforward gain through the loop is $\hat{\Gamma }$ (Fig 3A). Solving this recursion, we get (S1 Text):

$$p_{t}=f_{t}-\hat{\Gamma }\cdot \sum_{i=1}^{\infty }{\hat{\alpha }}^{i}f_{t-i}$$ (10)

Comparing Eq (10) to Eq (7), the feedback inhibitory network will implement the optimal linear prediction filter if

$$\hat{\alpha }=\beta \left(1-\Lambda^{*}\right)\;\text{and}\;\hat{\Gamma }=\Lambda^{*}/\left(1-\Lambda^{*}\right)$$ (11)

For the feedback inhibitory network (Fig 3B), we can identify and solve a similar pair of recursive equations (Methods). This gives us:

$$p_{t}=f_{t}-\frac{\Gamma }{1-\Gamma }\cdot \overset{\infty }{\underset{i=1}{\sum }}{\left(\alpha \cdot \left(1-\Gamma \right)\right)}^{i}\;f_{t-i}$$ (12)

where the interneuron’s discounting factor is *α* and the feedback gain is Γ. Comparing Eq (12) to Eq (7), the feedforward inhibitory network will implement the optimal linear prediction filter if:

$$\alpha =\beta \;\text{and}\;\Gamma =\Lambda^{*}$$ (13)

Summarizing the dependence of the optimal network parameters on the input statistics (from Eqs (11) and (13)) makes explicit the differences between the feedforward and feedback models in their adaptation to input changes:

**Table:** 

	Interneuron discounting factor	Gain
Feedforward	$\hat{\alpha }\left(\tau_{s},\sigma \right)=\beta (\tau_{s})\cdot \left(1-\Lambda^{*}(\beta (\tau_{s}),\sigma )\right)$	$\hat{\Gamma }\left(\tau_{s},\sigma \right)=\frac{\Lambda^{*}\left(\beta \left(\tau_{s}\right),\sigma \right)}{1-\Lambda^{*}\left(\beta \left(\tau_{s}\right),\sigma \right)}$
Feedback	*α*(*τ*_*s*_) = *β*(*τ*_*s*_)	Γ(*τ*_*s*_, *σ*) = Λ^*^(*β*(*τ*_*s*_), *σ*)

Although the resulting linear prediction-error filter changes in the same way for both circuits, the mechanistic difference between the circuits places different demands on the interneurons. For example, consider the changes in response to increasing input SNR (*σ*), for a fixed correlation of the signal component (*τ*_*s*_). For the feedforward network, $\hat{\Gamma }$ increases without bound, while $\hat{\alpha }$ approaches 0. In contrast, for the feedback network, Γ approaches 1 (Fig 2B), while *α* remains fixed to *β*. Therefore, whereas in the feedforward network both the gain and the interneuron time constant must vary to maintain optimality, in the feedback network, only the feedback gain must vary.

Focusing on the interneuron discounting factor, in the feedforward case, the interneuron gets progressively faster as noise increases, as if the interneuron is reducing the time over which it averages the signal to obtain a prediction, given cleaner inputs (with less noise). However, in the feedback case, the interneuron averages over the same time scale, perhaps to provide a matched filter to select the specific correlated signal. This emphasizes the different roles for the interneuron within each circuit.

Further, this difference suggests that the feedback network may lend itself more readily to the construction of an automatically adapting nonlinear network–to respond to rapidly varying input SNR. One can imagine changing the output from one component of a circuit using a nonlinearity, as is necessary for adaptation of the feedback network. However, it would seem to be quite difficult to vary the time constant of a neuron, a cellular property, using a nonlinearity, as is necessary for adaptation of the feedforward network. Therefore, we now explore the construction of such a nonlinear feedback circuit.

### Constructing a Nonlinear Circuit that Automatically Adapts to Changes in Input SNR

As introduced earlier, a nonlinearity can allow an invariant circuit to automatically change its linearized response to varying inputs [29–33]. Here, we consider the automatic adaptation of a feedback circuit to rapid changes of the input SNR by introducing such a nonlinearity. In the following sections, we demonstrate that its performance is close to optimal.

Our analysis of the optimal linear feedback circuit shows that as *σ* varies from 0 (pure noise) to ∞ (pure signal), the feedback gain, Γ, must increase from 0 to 1. Therefore, to automatically match the optimal filter over a range of input SNRs, the nonlinearity in the feedback inhibitory circuit must increase the strength of the output from the interneuron onto the principal neuron as the noise decreases (and vice versa).

Since inputs of different SNR are integrated differentially by the interneuron, we define the shape necessary for the static nonlinearity. Integrating uncorrelated noise is equivalent to a random walk. In contrast, integrating a correlated signal is equivalent to a biased random walk. Hence, on average, the output of a leaky integrator neuron, from an input with greater correlated component (i.e. higher SNR), will be larger in amplitude. Therefore, any automatic adapting nonlinearity–applied to the output of the feedback interneuron–must push the gain towards 0 for small output amplitudes, and pull the gain towards 1 for large output amplitudes.

**Fig 4:**
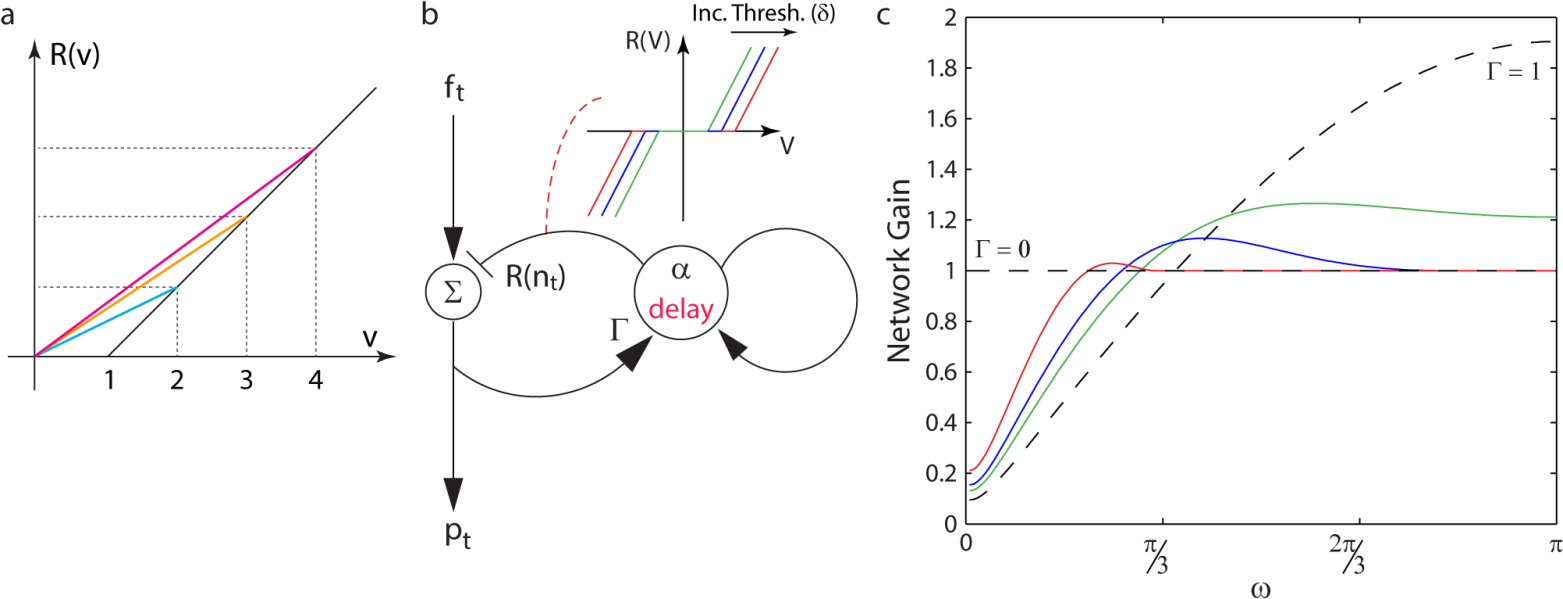
Rectification nonlinearity and its effect on the feedback inhibitory circuit. (a) Rectification nonlinearity (black), with threshold at v = 1. Linearized responses in color (cyan: min; magenta: max). (b) Nonlinear feedback inhibitory network. The nonlinearity (inset) is applied to the interneuron’s output. Nonlinearities with increasing thresholds are colored (green: min; red: max) (Methods). (c) Network gain at different input frequencies (note: the frequency space is in Z-space, i.e. defined with respect to the fixed time step of the network). The three colored curves are the nonlinear network response curves (computed using describing function analysis, colored as in (b)) (Methods). The dotted lines provide extreme parameter values for the linear network: Γ = 0,1.

One simple piecewise linear nonlinearity satisfies this requirement: the threshold or rectilinear nonlinearity, which increases linearly, from a fixed threshold (Fig 4A and 4B; Methods). In response to increasing input amplitudes, the linearized feedback gain of the nonlinear circuit increases from a gain of 0, for small inputs (below the threshold), to a gain of 1, for large inputs (colored lines in Fig 4A). This precisely matches the range over which Γ must vary, as the SNR of the input changes to minimize the network gain.

Also termed a dead-zone nonlinearity by engineers [45], the rectification nonlinearity is biologically plausible. The nonlinearity (Fig 4B) needs to respond symmetrically around 0, i.e. to both positive and negative inputs. This is biologically unreasonable for any one neuron. However, such a nonlinearity can be constructed by using a pair of neurons, with each receiving half the inputs (a single sign), and having oppositely signed output connections. The rectification can then be implemented through half-wave rectification of each of the neurons’ outputs, either through the neurons’ spiking thresholds, or through the minimum voltage required to release a vesicle [43,44].

Therefore, a feedback inhibitory circuit with a rectification nonlinearity applied to the feedback interneuron (Fig 4B) seems to be a plausible candidate to perform automatic adaptation, and approximate the minimal network gain, across a range of input SNRs in real neural circuits. To confirm this, we first demonstrate that the nonlinear feedback inhibitory network does, indeed, change its responses as the input properties change, and that the resulting network gain has the qualitative structure necessary for automatic adaptation.

### Qualitative Understanding of Nonlinear Network Dynamics

To understand the operation of the nonlinear feedback circuit (Fig 4B), independent of the specific value of the threshold, it is convenient to consider its network gain in the frequency domain (Fig 4C). This will provide intuition for the circuits’ response to inputs with different degrees of predictability. Indeed, since low frequency inputs change slowly, they are predictable. In contrast, high frequency inputs (near the Nyquist frequency) are unpredictable and, therefore, for the purposes of the feedback circuit equivalent to noise. The optimal linear feedback circuit for each of these input regimes differs. For low frequency (predictable) inputs, the optimal linear feedback circuit would set the feedback gain to 1. In contrast, for high frequency (unpredictable) inputs, the optimal linear feedback circuit should set the feedback gain to 0.

We observe that, without changing any parameters, each nonlinear network (with a specific threshold) shows network gains that approach those of the Γ = 1 linear network for low frequency inputs, and those of the Γ = 0 linear network for high frequency inputs (Fig 4C, dotted lines show the performance of the two linear networks). This suggests that the rectilinear feedback inhibitory circuit approaches the optimal performance in different regimes of activity, without internal adaptation, i.e. performance of the form necessary for automatic adaptation. Further, the performance is qualitatively independent of the precise value of the threshold, suggesting–as introduced above–the dynamically changing performance is a general property of the shape of the rectilinear nonlinearity.

We now demonstrate that this qualitative understanding is also supported quantitatively, for fast varying input statistics: comparing the performance of the nonlinear feedback network against that of optimal linear networks.

### Quantitative Performance of Nonlinear vs. Linear Circuits

To compare the quantitative performance of the nonlinear and the linear circuits in the regime where input properties change too fast to allow for parameter adaptation, we define a class of non-stationary inputs. Each such input–termed a mixture–is composed of two components with different SNRs, mixed in time, such that there is one component for a fixed amount of time, and then the second component for the same amount of time. In this way, we are modelling the response of the circuit to an input with a rapid change from one SNR to another, as opposed to a single input, with a fixed SNR.

Within this input regime, we compare the nonlinear network to two different types of linear networks:

- Type 1: The linear network that obtains the minimal network gain over the specific mixture of two SNR inputs, i.e. the minimal network gain for a non-adapting linear predictive coding network.
- Type 2: The linear network that has sufficient time to adapt separately to optimally transmit each component of the mixture, i.e. this network has the minimal network gain for any linear predictive coding network (over this specific input mixture).

**Fig 5:**
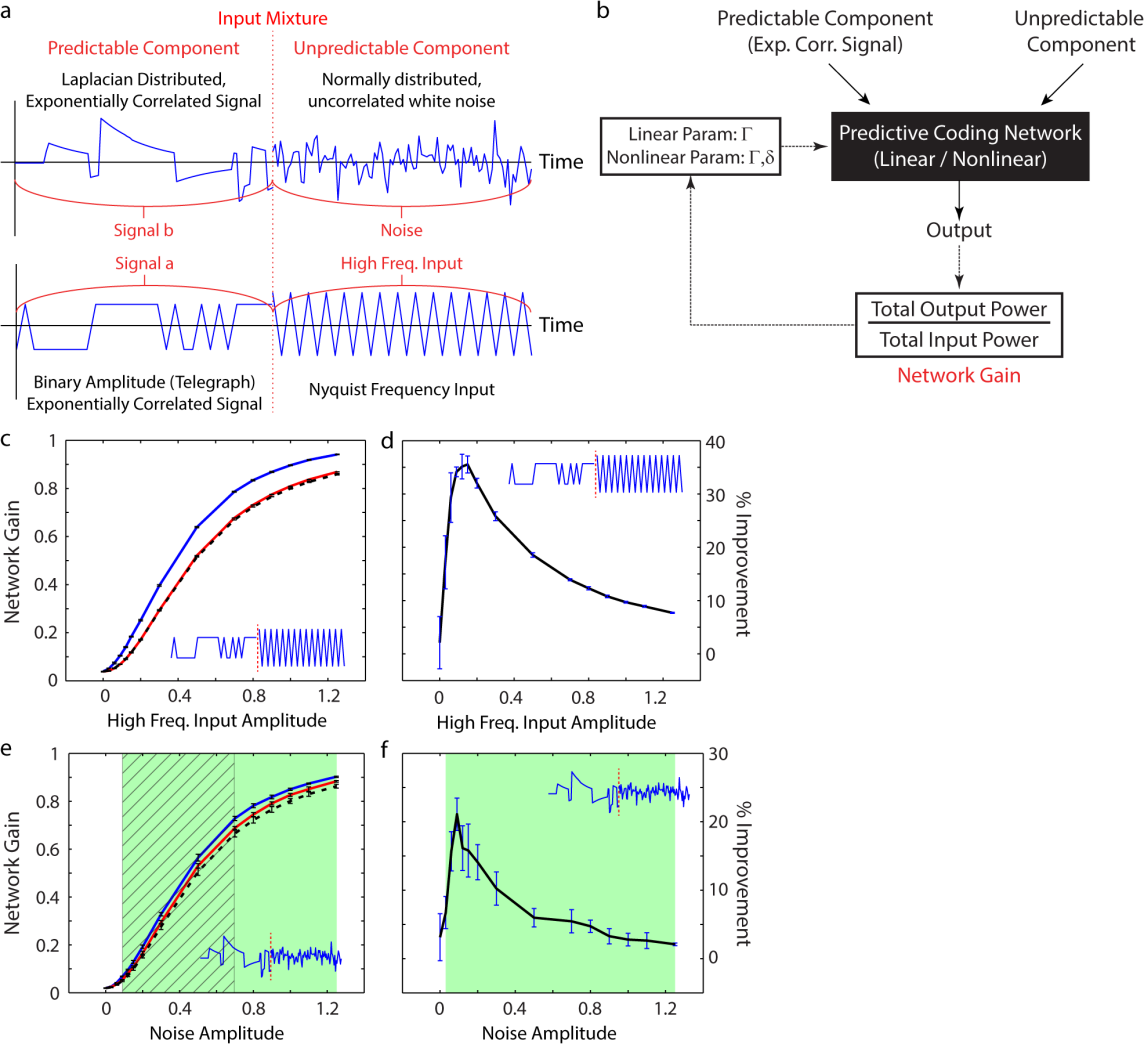
Comparing the performance (measured through network gain) of nonlinear and linear feedback inhibitory networks (measured through network gain: lower is better) (details in Methods). (a) Two input mixtures, modeling rapid transition from predictable to unpredictable input components. (b) Description of simulations. Inputs constructed as in (a). At each time point, inputs are either pure predictable signal, or pure unpredictable noise, with an instantaneous transition from one type to the other, in the middle of the simulation period. (c-f) Simulation outputs (inputs shown in inset). The amplitude of the unpredictable component of the mixture varies along the x axis. Error bars are 1 std. dev. (c,e) Network gain of the linear network of type 1, optimized to the mixture (blue, non-adapted linear response), is significantly higher than that of the nonlinear network (red). In contrast, the nonlinear network gain is close to the response of the optimal linear network of type 2, which is allowed to adapt to each component of the mixture (dotted black, adapted linear response). (e) Green shading indicates region where the nonlinear response is more than one std. dev. lower than the non-adapted linear response. Diagonal hashing indicates region where the nonlinear response is within one std. dev. of the adapted linear response. (d,f) % improvement of the performance of the nonlinear network over the type 1 linear network at different amplitudes of the unpredictable component. Data taken from (c) and (e) respectively. (f) Green box indicates region where the improvement is more than one std. dev. different from 0.

We demonstrate (Fig 5C and 5D) that the nonlinear network (red curves) both: (i) outperforms the type 1 linear network (blue curves), and (ii) approximates the performance of the type 2 linear network (dashed black curves).

In more detail, the response of both the linear and nonlinear networks to a mixture of two input components (Fig 5A) is simulated, and the network gain computed (summarized in Fig 5B). By varying the network parameters, we find the network which minimizes the gain for the specific mixture (Methods).

To robustly test the performance of the nonlinear circuit, we simulated its response to a mixture composed of components that are as distinct as possible. Therefore, we chose the first component of the mixture to be pure, predictable, correlated signal, and the second component to be unpredictable. As defined earlier, the correlated component is exponentially correlated with a fixed time constant. For the unpredictable component of the mixture, we utilize one of two inputs: (1) input at the Nyquist frequency, or (2) Gaussian white noise. Both these inputs are–for the purposes of a nonlinear predictive coding circuit with a non-zero time constant in the feedback neuron–unpredictable. Since the input mixture transitions from one extreme SNR to another, it should provide a strong test of the ability of a fixed nonlinear circuit to respond to a range of input SNRs.

We first show that the best linear network of type 1 is outperformed by the nonlinear network (Fig 5C, and green region in Fig 5E). Indeed, when the unpredictable component of the mixture was composed of input at the Nyquist frequency, the improvement of the nonlinear network over the type 1 linear network was particularly large (30–40%) (Fig 5D). When the unpredictable component of the mixture was modelled by the more biologically realistic white noise stimulus, the improvement was smaller, but it was still approx. 20% (Fig 5F).

It is important to note that the linear network of type 1 against which we compare the nonlinear network’s performance has the minimal network gain of any such network. We could have used a linear network adapted to the first component of the mixture, and then measured its performance over both components. This would be a natural model for the case where a network was adapted to some input statistic, which changed rapidly, and the network had had insufficient time to adapt to the new statistic. However, the type 1 linear network outperforms any such linear network. Therefore, it provides a strong baseline against which to compare the performance of the nonlinear network.

Our results show that the nonlinear network’s improvement over the type 1 linear network persists even if (a) the unpredictable component has larger average amplitude than the predictable (correlated) component (Fig 5C–5F), or if (b) the fraction of non-stationarity within the input is low (S2 Fig). In both cases, the greatest relative improvement occurs when the uncorrelated component is comparatively smaller (either in amplitude or time) than the correlated component. However, the improvement persists over a wide range of input mixtures. Therefore, mixtures of noisy inputs with cleaner ones can be dealt with automatically–and effectively–by the nonlinear network, independent of either the amplitudes of each component, or the amount of each component within the mixture (modelling the sampling distribution over the environment).

Continuing beyond the improvement over type 1 networks, we next observe that the performance of the nonlinear network approximates the performance of the type 2 linear network (Fig 5C and diagonal hashed region in Fig 5E). The type 2 linear network is allowed to adapt independently to each component of the mixture. Therefore, its performance is the lowest possible network gain, for any linear predictive coding algorithm–assuming sufficient time to adapt to each new input SNR. The observation that the nonlinear network is able to approximate the type 2 linear performance–despite having a fixed set of network parameters–for a range of input mixtures (with different input signal distributions), is precisely the desired, quantitative, demonstration of automatic adaptation.

Given this, we explore the potential role of nonlinear feedback inhibitory networks in real sensory systems.

### Comparison with Biological Experiments

Classical experiments, such as the seminal work of J. D. Victor in cat retinal ganglion cells [17], have shown that the adaptation of neural responses to varying input stimuli occurs nonlinearly. However, these observations do not directly assess the presence of an automatically adapting circuit since, classically, the neuronal responses were measured after the input had been introduced stably, for some time. Therefore, any observed nonlinear adaptation can be explained by the presence of a second, nonlinear estimate of the input statistics, whose output is then, secondarily, used to adapt the measured linear neural response properties. Such a mechanism would have a delay but, given the experimental time scale, such a delay was not constrained. Nevertheless, simulating the responses of the nonlinear feedback inhibitory network shows that its responses do agree qualitatively with the classically observed nonlinear experiments (S3 Fig).

**Fig 6:**
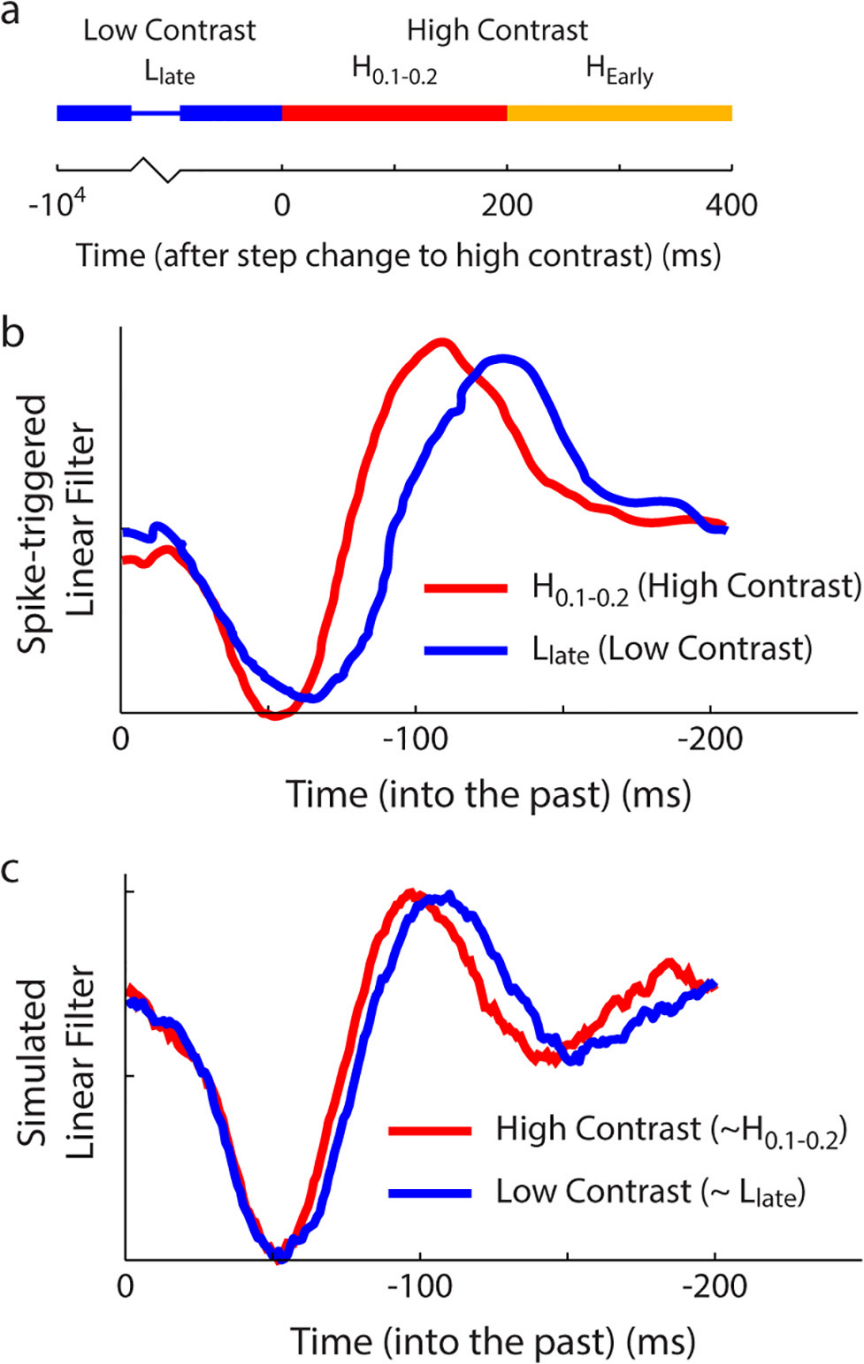
Linear filters from spike-triggered correlation analyses, estimated at different times around a sudden shift from low (L) to high (H) contrast. (a,b) Adapted from [27]. (a) Timeline of experiment. (b) Experimentally measured filters (low contrast, blue; high contrast, red). High contrast filter is computed only from the response to the first 200 ms of high contrast stimulus (see timeline in (a)). (c) Simulated response filters of the principal cell of the nonlinear predictive coding circuit (Methods), showing oscillatory structure.

**Fig 7:**
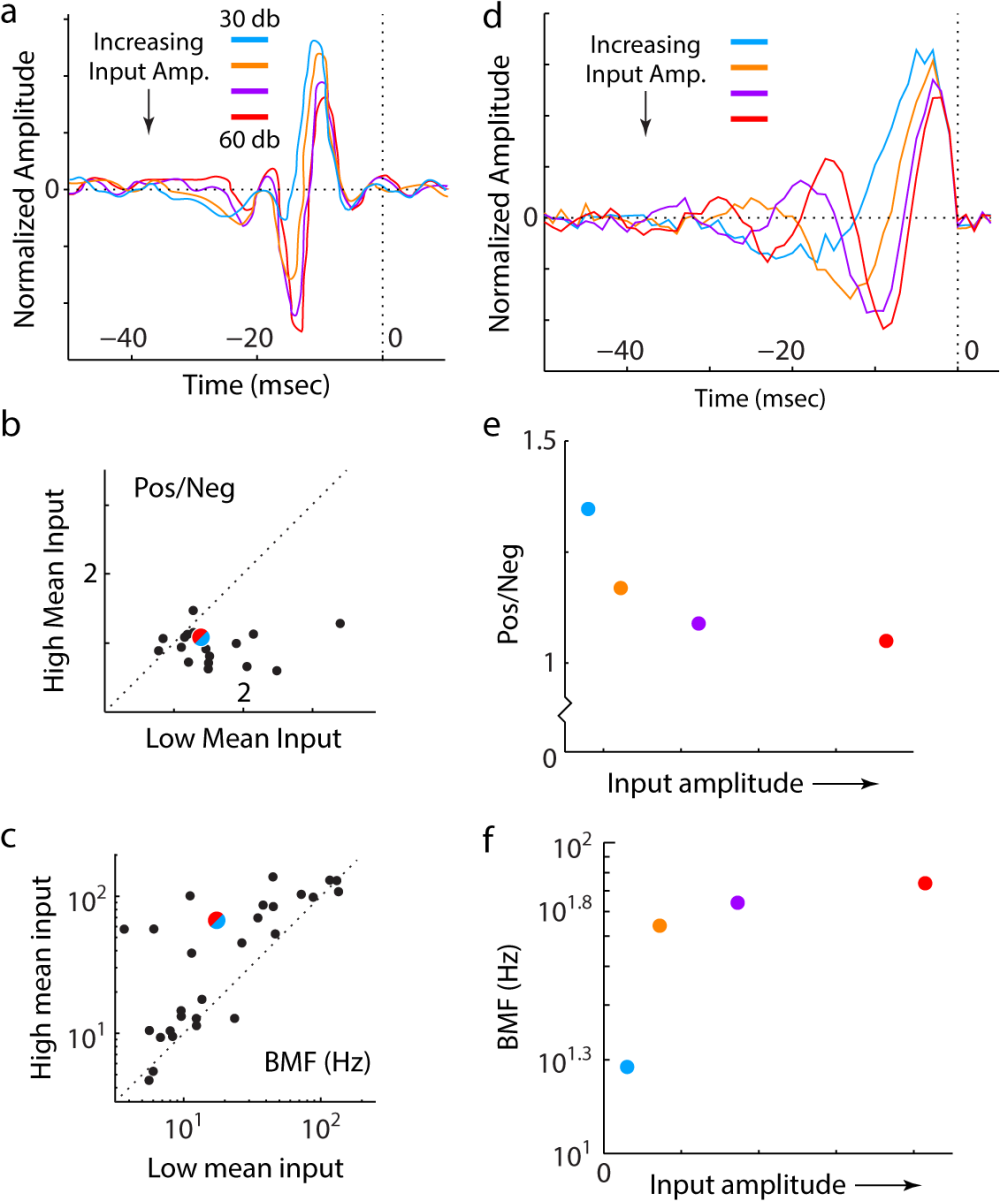
Response filters of zebra finch auditory neurons ((a), adapted from [28]) compared to theoretically simulated response filters of a nonlinear feedback inhibitory circuit (d). (a-c) Adapted from [28]. (a) Linear response filters for a single neuron stimulated with inputs of different mean sound amplitudes (colored to match (d)). (b) Ratio of the total positive to total negative component of neuronal filters is computed for an input with high mean amplitude, and an input with low mean amplitude. Each circle shows these values for a different recorded neuron (one example being (a)). The colored circle is derived from the simulated response filters of the principal cell of the nonlinear circuit (d). It uses the ratio of the total positive to total negative component of the simulated filters (plotted in (e)) at the highest input amplitude (red), and the lowest input amplitude (cyan). (c) The BMF (freq. of the peak of the Fourier transform of the linear response filters) for a high mean input, and a low mean input (for different neurons, one example being (a)). The colored circle is derived from the simulated responses of the principal cell of the nonlinear network (d). It uses the BMFs (shown in (f)) of the simulated filters at the highest input amplitude (red) and the lowest input amplitude (cyan). (d) Linear filters estimated to best approximate the response of the principal cell of the nonlinear circuit to white noise stimuli of different amplitudes (Methods). Curves colored to match (a). (e) The ratio of the total positive component of the curves in (d) to the total negative component. Points are colored as in (a,d). The ratios derived from inputs with highest (red) and lowest amplitude (cyan) define the colored circle in (b). (f) The BMF of the responses filters from (d). The BMF values derived from inputs with highest (red) and lowest amplitude (cyan) define the colored circle in (c).

To test specifically for the presence of automatic adaptation, experiments must constrain the speed of the change of the neuronal response function: an automatically adapting circuit will respond to a change in the input structure with an adaptive change, on the timescales of neuronal dynamics. Experimental evidence demonstrating these fast changes to neuronal responses has been found, recently, both in the salamander visual system [21,27] (Fig 6A and 6B) and in the auditory centers of the avian brain [28] (Fig 7A–7C). Therefore, these changes appear to be a general property of sensory systems. We demonstrate that these experimental changes match the expected changes in the response filters, within the nonlinear network.

Baccus and Meister [27] found that the linear filters of retinal ganglion cells (RGCs) changed as fast as could be experimentally measured–within 200ms of a sudden input change. Further, as the mean input contrast is increased, the filter shifts its maximum weights onto inputs from the more recent past (Fig 6A and 6B) [27].

To match the qualitative structure of the observed temporal filter, with a smooth increase from zero to the first peak, we make two biologically reasonable changes to the nonlinear model (Methods, S4 Fig).

1. We introduce an additional neuron with a non-zero time constant, prior to the principal neuron (S4B Fig) (modelling the upstream circuitry as a low pass filter, a first-order approximation).
2. We added a time constant to the principal neuron (S4C Fig) (since no real neuron can have a time constant of 0).

This modified network produces a smoothly varying temporal filter (with zero weight at t = 0) that can be compared to experiment (Methods). These two (biologically reasonable) changes are actually necessary; subsets of this model, with fewer neurons, or fewer time constants, won’t result in a smooth temporal filter (Methods).

Given this model, we demonstrate that the resulting response filter for the nonlinear feedback network shifts in the same direction as that measured by Baccus and Meister [27], in response to sudden input mean changes (Fig 5C). For example, the filter shifts towards more recent inputs (i.e. speeds up) in response to a sudden increase in input amplitude. This qualitative agreement between the change in the response filter introduced automatically by the nonlinear feedback network, and the responses of the RGCs, suggests that a nonlinear circuit could underlie the observed fast adaptation.

Mante et al. [21] also showed a rapid shift of the linear impulse response function for neurons in the lateral geniculate nucleus (LGN). Their observed change (Figures 3 and 4 in [21]) has the same qualitative structure as that observed by Baccus and Meister [27] (Fig 6B), but is observed in response to both changing input contrast and changing input luminance. Since the shift in the filter due to the nonlinear feedback inhibitory network is independent of the specific input, the nonlinear network is able to model the observed filter changes in the LGN.

This independence on the precise input also allows the automatic adaptation of the nonlinear feedback inhibitory network to generalize to non-visual sensory modalities. Nagel and Doupe [28] measured changes to the linear filters of auditory neurons in the zebra finch forebrain, which occurred rapidly, within 100ms of changes to the input auditory stimuli (Fig 7A). They quantitatively characterized these changes:

1. In response to increasing input amplitude, the first peak of the temporal filter decreased in amplitude, and the first valley increased in amplitude. Therefore, there was a decrease in the ratio of total positive response of the filter to total negative response, when the input amplitude changes from high to low (points below the diagonal in Fig 7B).
2. The shift in the location of the peaks can be characterized by the change in the peak frequency response of the filter (best mean frequency, BMF). Therefore, comparing high to low amplitude inputs, the authors identified an increase in the BMF (points above the diagonal in Fig 7C).

We demonstrate, through simulating the responses of the nonlinear feedback circuit (Fig 7D) that the shift in the filter responses agrees qualitatively with the shift in the measured linear responses (Fig 7A). Indeed, as the amplitude increases, one observes not only a shift in the locations of the extrema, but also the addition of a second peak, following the valley, at the highest amplitudes. Further, each of the changes to the filters measured by Nagel and Doupe [28] are a natural property of the nonlinear circuit:

1. The positive/negative ratio of the simulated filter of the nonlinear network decreases as the input amplitude increases (Fig 7E). Further, the simulated ratio at high and low input amplitudes agrees quantitatively with Nagel and Doupe’s measurements (colored circle in Fig 7B).
2. The BMF of the simulated temporal filters of the nonlinear network increases for increasing input amplitudes (Fig 7F). Further, the value of the BMF for the simulated filters at high vs. low input amplitudes agrees quantitatively with the experimental measurements (colored circle in Fig 7C).

This suggests that a nonlinear feedback circuit could underlie the observed fast adaptation in the zebra finch auditory forebrain.

Importantly, the changes to the response filters of the nonlinear predictive coding network are a general property of the network, and not a function of the specific parameters chosen. Indeed, it is possible to demonstrate analytically that a nonlinear model of neurons with just two time constants, assuming only that the time constant of the interneuron is longer than that of the principal neuron, already shows a shift of its single extremum towards the more recent past (as input amplitudes increase) (S1 Text). Therefore, the rectified feedback inhibitory circuit design automatically lends itself to fast changes of the response filters of the output neuron, matching the observed fast adaptations in many different neural circuits.

## Discussion

Neuronal circuits must transmit input signals which vary, rapidly, by multiple orders of magnitude [2,3,25,26], through neuron channels that have limited dynamic ranges and slow response times [1–5]. This results in two computational challenges: transmitting signals with minimal power, and responding to rapid changes in the input statistics. We demonstrated that an optimal predictive coding algorithm, that reduces the transmission power of a correlated signal (and thereby ameliorates the first challenge), can be implemented with linear leaky integrator neurons using either feedback or feedforward inhibition. Inclusion of a static nonlinearity in the feedback inhibition circuit allows it to approximate the performance of the optimal linear predictive filter, for a range of input SNRs, while keeping its circuit parameters unchanged. Such a circuit, therefore, helps to address the second challenge. Indeed, we showed that the nonlinear feedback inhibitory network’s responses are in agreement with experimentally measured rapid changes to neuronal response functions in different sensory modalities [21,27,28].

Our analysis distinguishing the feedforward and feedback implementations of the algorithm demonstrated the importance of the neural implementation in developing intuition about an algorithm. For example, in the feedforward inhibitory circuit, it was possible for predictive coding to be implemented by a neuron with a short time constant, for large SNR inputs. However, in the feedback circuit, the predictive neuron is matched to the properties of the signal component, and independent of the SNR. Therefore, each implementation may have differing properties, which may each prove differently useful.

This work also demonstrates the necessity of studying inputs with rapidly varying statistics, to understand the different constraints they place on circuit implementations of an algorithm. Both the feedforward and feedback circuits can implement optimal linear predictive coding–when the input is statistically stationary. However, by analyzing the responses of the two different implementations to non-stationary inputs, we found that the feedback implementation provides a natural way to approximate an optimal response, through the addition of a circuit nonlinearity. In contrast, the alternative, feedforward, implementation requires adaptation of its underlying cellular properties, which would be difficult to vary through a circuit nonlinearity.

In this report, we introduced intuition on the shape of a nonlinearity necessary to perform automatic adaptation. Given its mathematical convenience, and biological plausibility, we focused on the rectilinear nonlinearity. However, it is important to note that the rectification nonlinearity is not the only nonlinearity that can satisfy the necessary structure. Indeed, any nonlinearity with the necessary inflection point should also be able to perform an automatic adaptation. This provides an avenue for further analysis.

Another avenue for further exploration is how to generalize our results on nonlinear predictive coding networks to more complex stimuli. We found the optimal linear predictive coding algorithm for an exponentially correlated signal with a single time constant. However, naturalistic stimuli can be modeled as a combination of several exponentially correlated signals, with different time constants. This suggests that to respond to a naturalistic stimulus, there should be several predictive coding circuits, each adapted to one of the correlations within the signal. However, how could these different predictive coding circuits be combined to respond optimally overall? One possible solution may be the addition of mutually inhibitory connections between the parallel predictive coding circuits. This circuit design should allow each neuron to respond maximally to the input component that it was adapted for, while simultaneously removing that input component from the remaining neurons. Hence, it might allow the net response of the larger circuit to remain close to optimal. A similar network design has been shown to implement predictive coding across a spatial scene (for a non time-varying stimulus) [46]. Exploring circuits of this form, built using simpler building blocks, should prove useful in understanding the design of more complex circuits.

Another direction to explore is the computational function of the nonlinear circuit, beyond its linearized responses. In this work, we demonstrated that the network gain of the nonlinear feedback network approximates that of the optimal linear network. However, this does not imply the two networks have identical responses to stimuli. For example, the linear algorithm amplifies high frequency inputs (flattening the output frequency distribution, for an exponentially correlated signal with noise) (S5 Fig). In contrast, the nonlinear network reduces the transmission of high frequency inputs (since they are less likely to cross the threshold). This difference, where the nonlinear network may partially denoise the input, hints at additional, computational functions for nonlinear networks, which should be explored further.

In general, adaptation of network dynamics, causing them to respond faster when inputs are more salient, has been observed in different experiments [2,12,17,27,28], often as a shifting of the linear kernels. Conceptually, this shift has been understood as the system responding more quickly to salient stimuli, since the relevant information can be extracted sooner [47]. Our results suggest that nonlinear feedback inhibitory circuits may provide a general neural mechanism with which to implement such a shift, automatically. In addition, the presence of fast adaptation in different species suggest that this mechanism may have been conserved over evolutionary history.

Finally, one long standing goal of computational neuroscience has been to develop circuit motifs, in a manner similar to electrical engineering. We believe that the nonlinear feedback inhibitory network could be one such neuronal circuit motif. It performs a specific computational function without losing information, and is stable with respect to internal disturbances (S1 Text). Hence, this nonlinear motif could be inserted into a larger computational circuit without affecting its function, while still maintaining stability, and lowering the internal dynamic range requirements. Further, the circuit’s structure is simple and biologically plausible. Hence, it can reasonably be implemented in many areas of the central nervous system. Therefore, we believe that the identification of this motif should provide a useful tool in the analysis of larger circuits in many neural systems.

## Methods

### Input Structure

The input used in optimizing the linear predictive coding circuit (as in Eq (3)) was composed of an exponentially correlated signal and uncorrelated noise, combined with an SNR (of the power of the input) of *σ*. In detail, the signal component of the input, *s*_*t*_, was defined as:

〈st〉=0〈stst+i〉=e−iτs≡βi (14)

and the noise, *ε*_*t*_, as:

$$\begin{array}{l} \langle \varepsilon_{t}\rangle =0\\ \langle \varepsilon_{t}\varepsilon_{t+i}\rangle =\delta \left(i\right)=\{\begin{array}{c} 1\\ 0 \end{array}\;\begin{array}{c} i=0\\ i\neq 0 \end{array} \end{array}$$ (15)

Our derivation of the optimal linear predictive coding filter did not require any constraint on the distributions (for either the signal or noise components of the input). Hence, for maximal generality, we left them unconstrained.

### Leaky Integrator Neurons

The predictive coding circuits were constructed with linear leaky integrator neurons (S1 Fig). The voltage dynamics of a single such neuron is described by:

$$\dot{v}\left(t\right)=-\frac{1}{\tau_{m}}v\left(t\right)+\frac{g_{s}}{\tau_{m}}v_{input}\left(t\right)$$ (16)

where the synaptic conductance, g_s_, is measured as a fraction of the cell's membrane conductance. Discretizing Eq (16) in time, and solving for v, we find that the resulting neuronal building block is an exponential, low pass filter, with time constant *τ*_*m*_, as described in the text.

$$v_{t}=\frac{g_{s}}{\tau_{m}}\sum_{i=0}^{\infty }e^{-\frac{i}{\tau_{m}}}v_{t-i}^{input}$$ (17)

### Expanding the Feedback Recursion

The steps to derive the recursive equation governing the dynamics of the feedback circuit Eq (12) are shown. The corresponding derivation for the feedforward circuit can be found in S1 Text.

The discretized feedback circuit is described by a pair of linear, recursive equations. As introduced in the text, we derive this pair of equations by computing the input to each cell in the circuit (Fig 3A), and adding a delay for all inputs to the interneuron. We can ignore the corresponding delay onto inputs to the principal cell, because adding an additional delay to the inputs to *p*_*t*_, i.e. having *p*_*t*_ = *f*_*t*−1_ − *n*_*t*−1_, would not change the structure of the recursion in Eq (9). It would simply add an additional time step of delay to everything.

This process gives us:

$$p_{t}=f_{t}-n_{t}$$ (18)

$$n_{t}=\alpha \left(n_{t-1}+\Gamma p_{t-1}\right)$$ (19)

Substituting Eq (18) into Eq (19) (at time point, t-1):

$$n_{t}=\alpha \cdot n_{t-1}+\alpha \cdot \Gamma \cdot \left(f_{t-1}-n_{t-1}\right)=\alpha \left(1-\Gamma \right)\cdot n_{t-1}+\alpha \cdot \Gamma \cdot f_{t-1}$$ (20)

Letting *η* = *α*(1−Γ), and then substituting Eq (20), at an earlier time point, back into itself, we get:

$$n_{t}=\eta \cdot \left(\eta \cdot n_{t-2}+\alpha \cdot \Gamma \cdot f_{t-2}\right)+\alpha \cdot \Gamma \cdot f_{t-1}=\eta^{2}\cdot n_{t-2}+\alpha \cdot \Gamma \cdot \left(f_{t-1}+\eta \cdot f_{t-2}\right)$$ (21)

Repeating this process, we have:

$$n_{t}=\alpha \cdot \Gamma \cdot \sum_{i=1}^{\infty }\eta^{i-1}\cdot f_{t-i}=\frac{\Gamma }{1-\Gamma }\cdot \sum_{i=1}^{\infty }{\left(\alpha \left(1-\Gamma \right)\right)}^{i}\cdot f_{t-i}$$ (22)

Finally, substituting back into Eq (18) gives the expanded recursion in the text, Eq (12).

### Rectilinear Nonlinearity

The equation for the rectilinear nonlinearity (also known as a dead zone nonlinearity in the engineering literature) is as follows:

$$R(x)=\{\begin{array}{ccc} x+\delta & & x<-\delta \\ 0 & & -\delta <x<\delta \\ x-\delta & & \delta <s \end{array}$$ (23)

### Describing Function Approximation of Nonlinear Network

In Fig 3B, we present the Bode plots of both the linear and the nonlinear networks. However, the Bode plot for the nonlinear network is, necessarily, an approximation (since the response of any nonlinear network is dependent on its input history). The approximation used in this plot is obtained through Describing Function analysis [45], and is commonly used in control theory.

In this analysis, we assume that the response of the network to a single frequency of input (at a single amplitude) is linear for each such input. This linear model is allowed to vary with each different frequency (and amplitudes). We compute a look-up table for the effect of the nonlinearity by balancing the input across the nonlinear loop, for each frequency (and input amplitude). For every single frequency input to the network, however, it is necessary to make the assumption that the output of the network only produces a single frequency output (the primary component of the Fourier transform). This means that describing function analysis automatically discards any spread of the initial frequency into higher Fourier harmonics. The resulting Bode plots are, therefore, not quantitatively correct. However, it has been well established that describing function analysis does provide a reasonable, qualitatively correct result.

### Comparison of Linear to Nonlinear Network Gains

Response of optimal linear and nonlinear networks to varying mixtures of stimuli was simulated. To construct these plots, a 1:1 mixture of two input components was used. The first component was pure exponentially correlated signal, and the second was pure noise. Further, the amplitude of the noise component was varied (values on the x-axis of Fig 5C–5F), while the signal amplitude was kept constant at 1.

The response of three different networks, two linear and one nonlinear, was simulated for each input mixture, and the network gains computed. For all three networks, the discounting factor was matched to the time constant of the input within the signal component of the mixture. Parameter variation was then used to find the optimal value of Γ (for the linear networks) and both Γ and the threshold *δ* (for the nonlinear network) that allowed the corresponding network to minimize the cost function. For the type 1 linear network, Γ was only optimized once, over the entire mixture. However, for the type 2 linear network, Γ was optimized twice, for each component of the mixture, separately. Finally, for the nonlinear network, the parameters were again only optimized once, over the entire mixture. The resulting network gains, and relative % improvements are plotted in Fig 5C–5F.

### Comparing Nonlinear Feedback Circuit to Experiment

It was necessary to modify the analytically-derived nonlinear feedback circuit to obtain simulations that can be directly compared to the experimentally measured response filters of different sensory neurons. The experimentally measured filters place a low weight on inputs at t = 0, with the weight increasing to a peak, followed by a reducing oscillation between peaks and troughs (Figs 5B and 6D). However, with the structure of the network introduced analytically (diagrammed in S4A Fig), it is straightforward to show that the response filter of the corresponding linear circuit applies a maximal weight at t = 0.

Since real neurons must have a non-zero time constant, the first change that we considered to the model, was to add a non-zero time constant to the principal neuron. However, the linearized response filter of this modified model still has maximal weight at t = 0. An alternative change is to add a time constant to a neuron providing input to the two-neuron network (S4B Fig). Since real neurons are embedded in a larger network of neurons, this is reasonable. However, again, the linearized response filter of this three-neuron model, with two non-zero time constants, has maximal weight at t = 0. In contrast, combining both the above changes into a single model with three neurons, all with non-zero time constants (S4C Fig), provides a model that shows the same behavior as experimentally measured, with a low weight on inputs from the immediate past, increasing to a peak, followed by a decrease to a trough. This model is a simple, biologically reasonable modification to the original predictive coding model that provides a response filter that is qualitatively similar to experiment. Therefore, we used this model for the in silico comparisons with experiment in Figs 5 and 6.

### Response Filter of Nonlinear Network for Inputs of Different Amplitude

The response of the nonlinear network, with three neurons with non-zero time constants (S4 Fig), was simulated for >2000 stimulus patterns, each containing 1000 time points of uncorrelated white noise. The stimulus amplitude was varied by varying the standard deviation of the sampling distribution. The linearized filter response of the nonlinear network to the inputs was then estimated by picking a point in time, and weighting the input to the network on each trial by the output of the network at that time point. This method is an analog of the spike-triggered averaging (STA) algorithm utilized by experimentalists (and is only modified by the use of the graded output values, in the absence of an output spiking neuron). The filter responses to inputs of different amplitudes could then be compared, and appropriate parameters extracted from the simulated curves.

## Supporting Information

S1 Fig Single compartment model of a linear neuron. Electrically, the neuron is an RC circuit, with inputs arriving as current (g_s_v_input_).
(TIF)

S2 Fig % improvement in network gains for a nonlinear network (over the corresponding linear network) while varying the fraction of the signal component to the noise component in a two component input mixture. Both the linear and nonlinear networks are only allowed to adapt to the mixture (and not to each individual component).
(TIF)

S3 Fig Nonlinear responses of X-type retinal ganglion cells (adapted from [17]) compared to simulated responses of the nonlinear feedback inhibitory circuit. (a) Firing rate of X-type retinal ganglion cells in response to a stimulus pulse of increasing contrast; dashed lines denote peak (cyan) and steady-state (red) responses. (b) Ratio of steady-state amplitude to peak amplitude for experimental (squares) and simulated model responses (diamonds). The reduction in the ratio, as measured experimentally, is qualitatively the same as the simulation. (c) Bode plots of responses of retinal ganglion cells, for sinusoidal stimuli with increasing contrast (figure adapted from [17]). As contrast increases, the peak frequency response increases in amplitude, and shifts to higher frequencies; similarly, the phase shifts rightwards. (b) Bode plots of the transfer function of the nonlinear predictive coding network, for increasing input contrasts (increasing from grey to black), computed using describing function analysis (Methods, [45]). The shifts observed are qualitatively similar to those measured experimentally (see (c)).
(TIF)

S4 Fig Modifications to the nonlinear feedback inhibitory network to allow comparison with experiment (Methods). (a) Nonlinear predictive coding circuit (as in Fig 4B). (1) Principal neuron; (2) Interneuron. (b) Additional neuron (3), upstream of predictive coding circuit (with non-zero time constant,χ). Model, without nonlinearity, used in analytical analysis of shifting response filters. (c) Neuron (1) modified to include a non-zero time constant. Model used in in silico simulations shown in Fig 6 and Fig 7.
(TIF)

S5 Fig Whitening effect of the optimal linear network for inputs with different SNRs. (a-e) Input power (blue) and the power within the optimal transfer function of the network (red) at different frequencies. SNR decreases from (a)–(e) (f-j) Output power at each frequency (obtained by multiplying both functions from left column). Notice the flat output power, termed whitening. Also, notice the reduction in total transmitted power. This reduction in power get progressively less as the fraction of predictable signal within the input reduces (i.e. as the SNR decreases). At the extreme case, in the final row, with pure noise, the input has the same power as the output, with no reduction of gain.
(TIF)

S1 Text Supplementary methods and proofs. (PDF)
