# Supplementary figures and images for: Automatic Adaptation to Fast Input Changes in a Time-Invariant Neural Circuit
Source: PLoS Comput Biol. 2015 Aug 6;11(8):e1004315. doi: 10.1371/journal.pcbi.1004315 (PMC4527762; doi:10.1371/journal.pcbi.1004315)

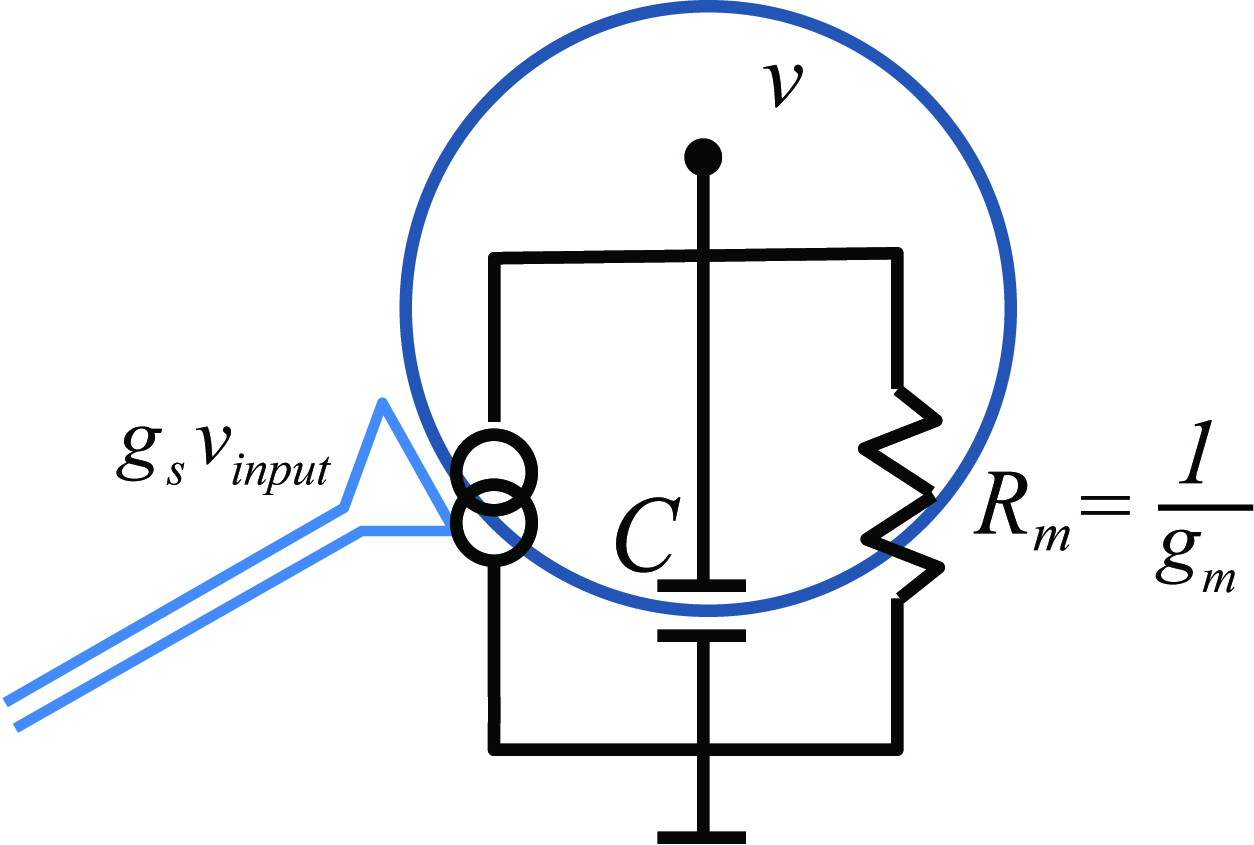

Supplement: S1 Fig — Electrically, the neuron is an RC circuit, with inputs arriving as current (gsvinput). (TIF) [file pcbi.1004315.s001.tif]

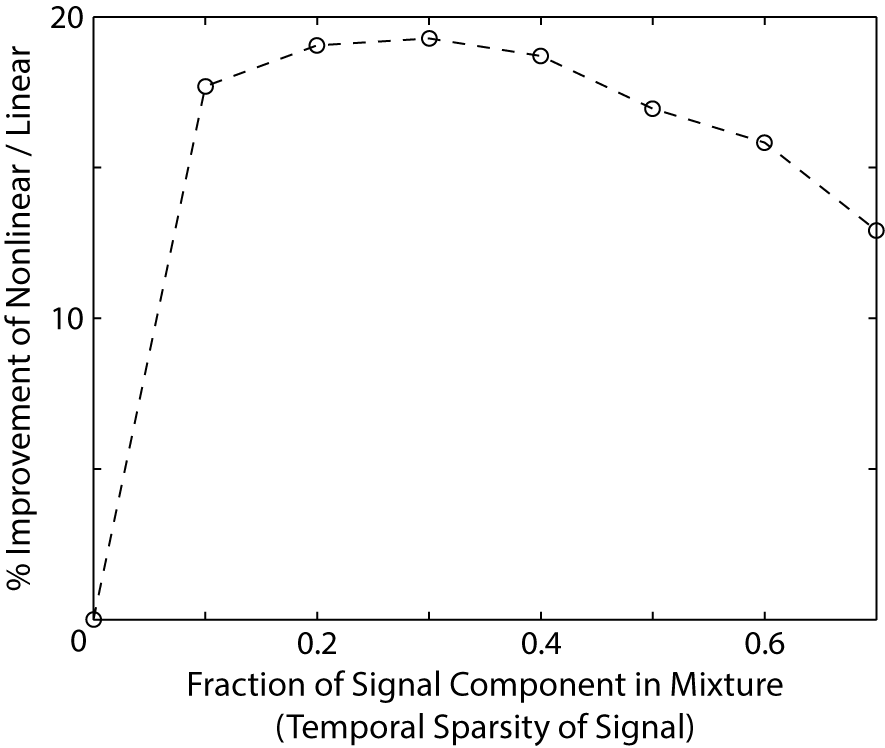

Supplement: S2 Fig — Both the linear and nonlinear networks are only allowed to adapt to the mixture (and not to each individual component). (TIF) [file pcbi.1004315.s002.tif]

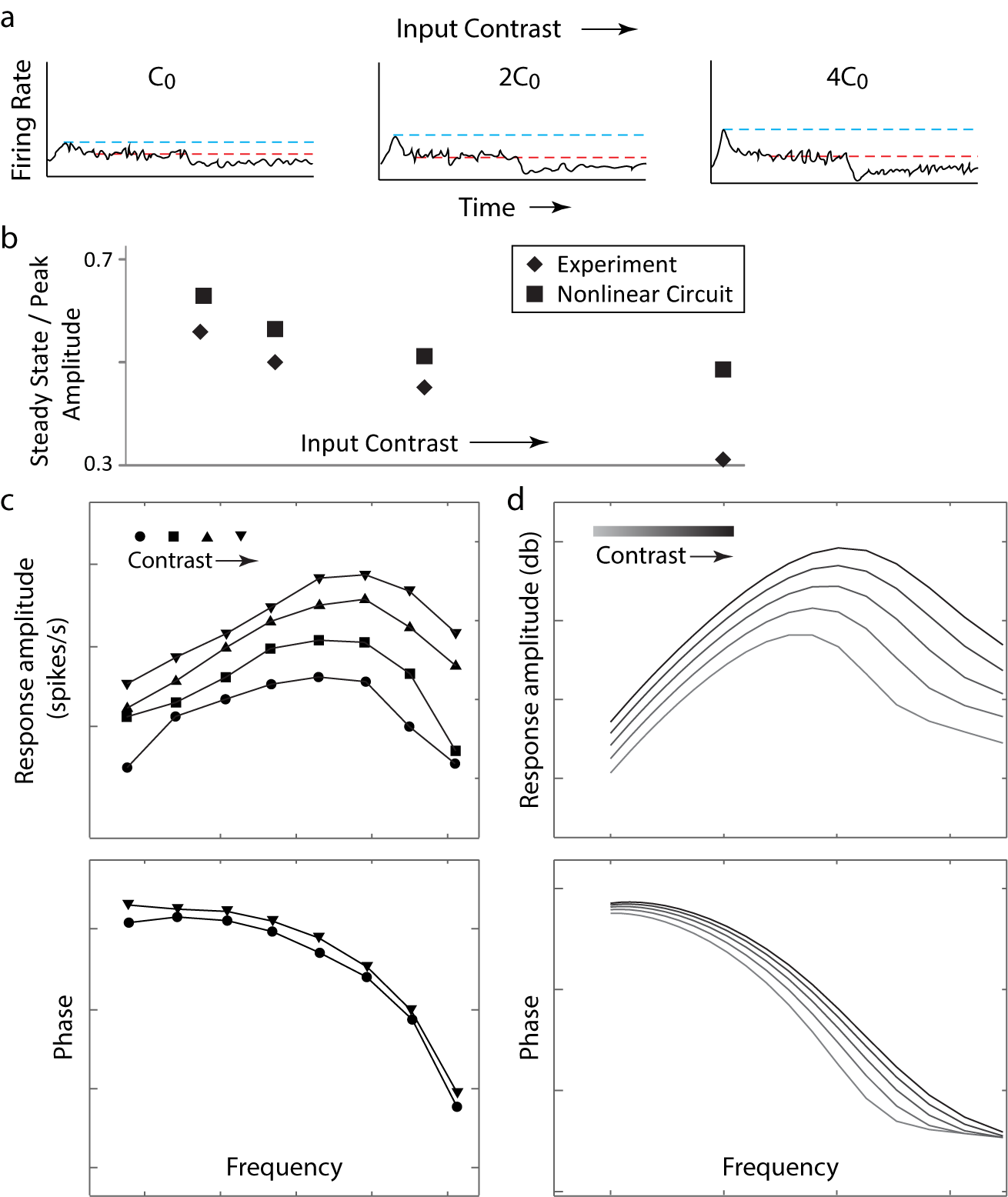

Supplement: S3 Fig — (a) Firing rate of X-type retinal ganglion cells in response to a stimulus pulse of increasing contrast; dashed lines denote peak (cyan) and steady-state (red) responses. (b) Ratio of steady-state amplitude to peak amplitude for experimental (squares) and simulated model responses (diamonds). The reduction in the ratio, as measured experimentally, is qualitatively the same as the simulation. (c) Bode plots of responses of retinal ganglion cells, for sinusoidal stimuli with increasing contrast (figure adapted from [17]). As contrast increases, the peak frequency response increases in amplitude, and shifts to higher frequencies; similarly, the phase shifts rightwards. (b) Bode plots of the transfer function of the nonlinear predictive coding network, for increasing input contrasts (increasing from grey to black), computed using describing function analysis (Methods, [45]). The shifts observed are qualitatively similar to those measured experimentally (see (c)). (TIF) [file pcbi.1004315.s003.tif]

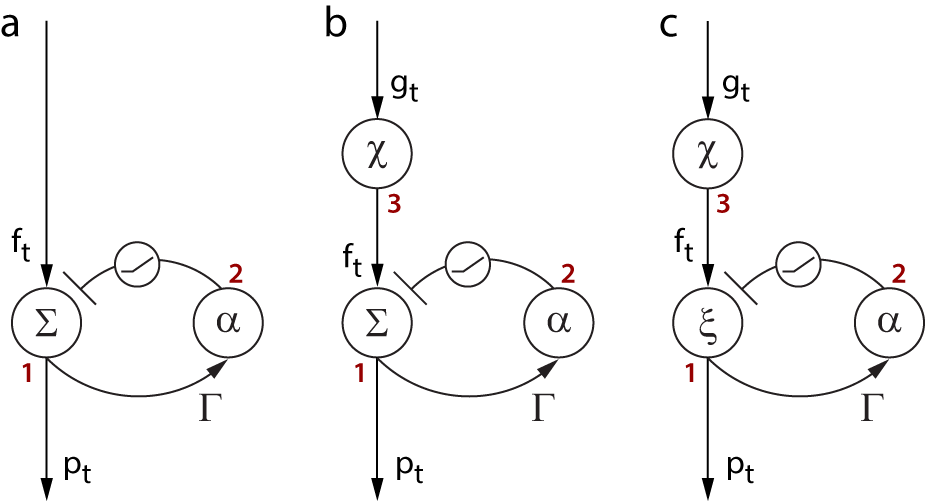

Supplement: S4 Fig — (a) Nonlinear predictive coding circuit (as in Fig 4B). (1) Principal neuron; (2) Interneuron. (b) Additional neuron (3), upstream of predictive coding circuit (with non-zero time constant,χ). Model, without nonlinearity, used in analytical analysis of shifting response filters. (c) Neuron (1) modified to include a non-zero time constant. Model used in in silico simulations shown in Fig 6 and Fig 7. (TIF) [file pcbi.1004315.s004.tif]

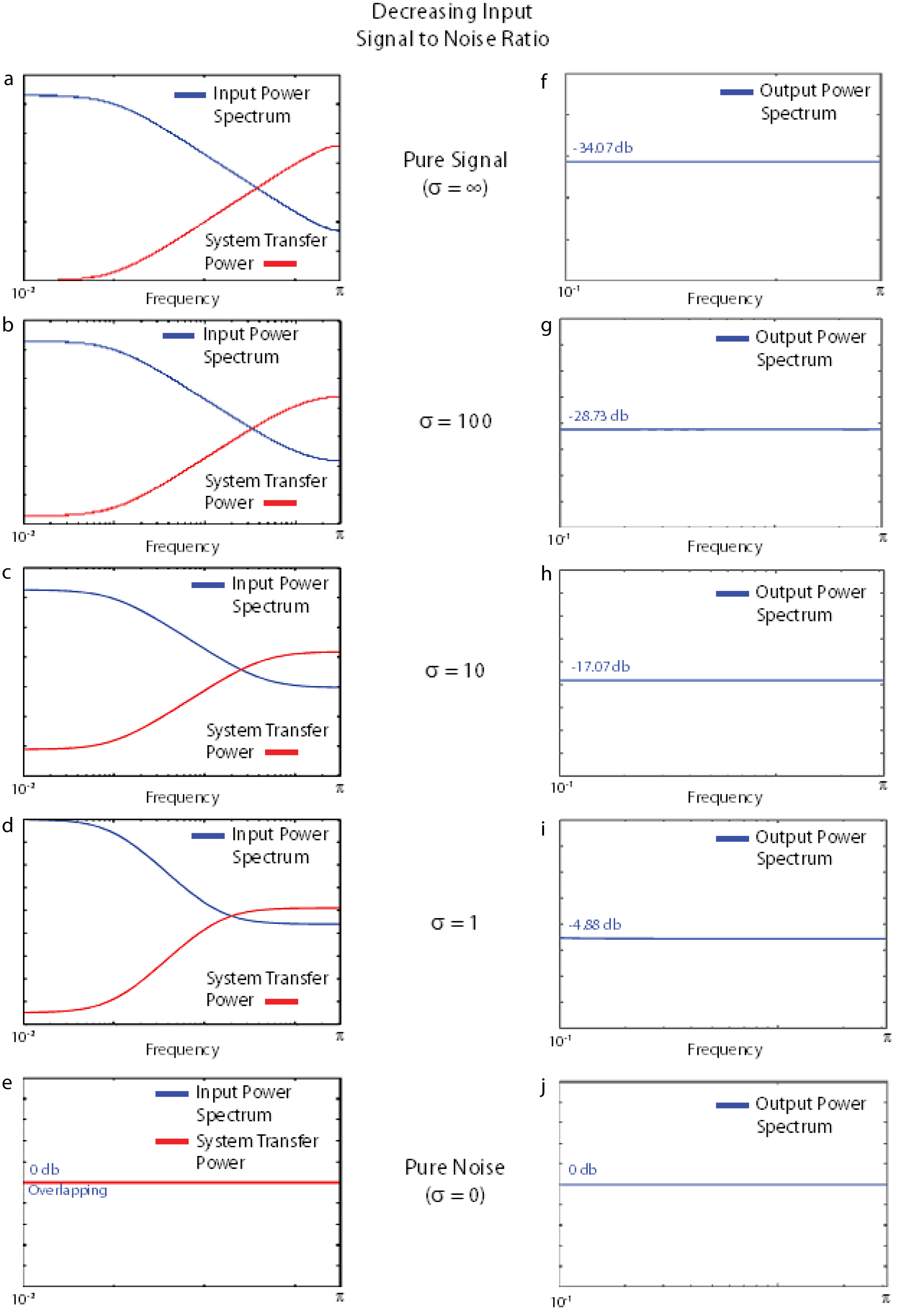

Supplement: S5 Fig — (a-e) Input power (blue) and the power within the optimal transfer function of the network (red) at different frequencies. SNR decreases from (a)–(e) (f-j) Output power at each frequency (obtained by multiplying both functions from left column). Notice the flat output power, termed whitening. Also, notice the reduction in total transmitted power. This reduction in power get progressively less as the fraction of predictable signal within the input reduces (i.e. as the SNR decreases). At the extreme case, in the final row, with pure noise, the input has the same power as the output, with no reduction of gain. (TIF) [file pcbi.1004315.s005.tif]
