# Supplementary material for: Automatic Adaptation to Fast Input Changes in a Time-Invariant Neural Circuit
Source: PLoS Comput Biol. 2015 Aug 6;11(8):e1004315. doi: 10.1371/journal.pcbi.1004315 (PMC4527762; doi:10.1371/journal.pcbi.1004315)
Supplement: S1 Text — (PDF) [file pcbi.1004315.s006.pdf]

Supporting Text

Text S1

Lossless reconstruction from a feedback predictive coding network

We show that it is possible to reverse a feedback predictive coding network, so as to reconstruct the input. (Note: we focus on the feedback predictive coding network, here, since that is the focus of our attention in the paper.)

Consider the recursive equations describing the dynamics of the network (where the predictor can be implemented linearly or nonlinearly).

$$\begin{aligned} p_t &= f_t - R(n_t) \\ n_t &= N(n_{t-i}, p_{t-i}) \end{aligned} \qquad \text{(A.3.1)}$$

where R() and N() are general functions (linear or nonlinear). Combining both equations in (A.3.1), we get:

$$p_t = f_t - R\left(N\left(n_{t-i}, p_{t-i}\right)\right) \qquad \text{(A.3.2)}$$

Solving for the initial input, we get:

$$p_t = f_t - R\left(N\left(n_{t-i}, p_{t-i}\right)\right) \qquad \text{(A.3.3)}$$

We can now define a reconstruction of the input, r, using (A.3.3).

$$r_t \equiv p_t + R\left(N\left(n_{t-i}, p_{t-i}\right)\right) \qquad \text{(A.3.4)}$$

It is clear that (A.3.4) depends only on the transmitted output, p_t, and functions thereof. Therefore, it is possible to reconstruct the input from the output of our feedback inhibitory predictive coding network alone, and, hence, the network transmits information losslessly.

Notice that the resulting reconstruction algorithm is stable, despite the necessary summation of two different components. Stability is only a problem for feedback networks, which can contain poles within their transfer functions [45]; in contrast, the reconstruction algorithm results in a feedforward network (Cƛ∃dzǊŜ мǀ·ǘ ,and hence errors cannot accumulate across multiple iterations of the loop.

Causality and optimal transmission

Assume that the predictive filter in Eq 2 were not causal. Then it would be possible to construct a temporal filter that has 0 network gain. However, such a filter would model the input perfectly – and, hence, would have high reconstruction error. Given that our choice of performance metric – network gain – is dependent on the having lossless reconstruction, it is therefore necessary that the predictive filter be causal.

Given that the filter in Eq 2 is indeed causal, a non-trivial temporal filter exists that has a minimal network gain. Indeed, the network gain has a lower bound given by zero, which can only occur if the predictive filter matches the input perfectly. This can never occur given a purely causal filter (since the first time point can never be predicted). Hence, the network gain must be strictly greater than 0, and some specific choice of temporal filter will result in a minimal network gain.

Optimal linear predictive coding algorithm

As introduced in the text, the solution of the optimal linear network has been, previously, solved in the adaptive signal processing literature. As such, it is possible to simply present the solution[8,10]. However, here, we derive the format of the optimization that was shown in the text, which is particularly convenient to implement with neuronal circuits.

We perform the optimization in the frequency domain. Since we are now working in the discrete time domain, with difference equations, we will use the Z transform. We can write out the linear difference equations that govern the dynamics of the network by following a single time step of input around the circuit:

$$p_t = -n_t + f_t$$
$$n_t = e^{-1/\tau}(n_{t-1} + \Lambda \cdot p_{t-1})$$

Setting $\alpha = e^{-1/\tau}$, we have:

$$p_t = -n_t + f_t \quad \text{(A.6.1)}$$
$$n_t = \alpha \cdot (n_{t-1} + \Lambda \cdot p_{t-1}) \quad \text{(A.6.2)}$$

Substituting (A.6.1) into (A.6.2), and taking a Z transform, gives us:

$$z \cdot n = \alpha \cdot \left(n + \Lambda\left(-n + f\right)\right) \quad \text{(A.6.3)}$$

Solving for n, and substituting back into (A.6.1) (which is unchanged by the Z transform):

$$p = \left(1 - \frac{\alpha \cdot \Lambda}{z - \alpha + \alpha \cdot \Lambda}\right) f$$
$$TF(z) \equiv \frac{p}{f} = \frac{z - \alpha}{z - \alpha\left(1 - \Lambda\right)} \quad \text{(A.6.4)}$$

Defining $b = \alpha\left(1 - \Lambda\right)$, we can simplify the notation:

$$TF\left(z\right) = \frac{z - \alpha}{z - b} \quad \text{(A.6.5)}$$

To compute the cost of transmitting a signal following the application of this transfer function, we compute the Z transform of the autocorrelation function of the signals of interest. This allows us to get the power of the input signal. For our input, composed of two components that are uncorrelated to each other, we compute the Z transforms for the signal and the noise independently, and then sum them together.

The autocorrelation function of the exponentially correlated signal is given in Eq 14. By definition (reference), the Z transform of the autocorrelation function is:

$$\sum_{n=-\infty}^{+\infty} e^{-|n|/\tau_s} z^n \quad \text{(A.6.6)}$$

Splitting the sum up:

$$\sum_{n=-\infty}^{-1} e^{-1/\tau_s} z^n + \sum_{n=0}^{+\infty} e^{-|n|/\tau_s} z^n$$

$$\sum_{n=1}^{+\infty}\left(\frac{e^{-1/\tau_s}}{z}\right)^n + \sum_{n=0}^{+\infty}\left(e^{-1/\tau_s} z\right)^n = \frac{1}{1-\frac{e^{-1/\tau_s}}{z}} - 1 + \frac{1}{1-e^{-1/\tau_s} z}$$

Further simplifying:

$$\frac{e^{-1/\tau_s}}{z-e^{-1/\tau_s}} + \frac{1}{1-e^{-1/\tau_s} z} = \frac{e^{-1/\tau_s}\left(1-e^{-1/\tau_s} z\right) + z - e^{-1/\tau_s}}{\left(z-e^{-1/\tau_s}\right)\left(1-e^{-1/\tau_s} z\right)}$$

$$\frac{z-e^{-2/\tau_s} z}{\left(z-e^{-1/\tau_s}\right)\left(1-e^{-1/\tau_s} z\right)} = \frac{z\left(e^{1/\tau_s} - e^{-1/\tau_s}\right)}{\left(z-e^{-1/\tau_s}\right)\left(e^{1/\tau_s} - z\right)}$$

Using the variable β, we have:

$$\Phi_1(z) = \frac{z\left(1-\beta^2\right)}{(z-\beta)(1-\beta z)} \quad \text{(A.6.7)}$$

Now, doing the same for the uncorrelated white noise, we have:

$$\sum_{i=-\infty}^{+\infty} \delta(n) z^n \quad \text{(A.6.8)}$$

The discretized version of the delta function has value 0, for every input, n, except at 0. Therefore:

$$\Phi_2 = \sum_{n=-\infty}^{+\infty} \delta(n) z^n = z^0 = 1 \quad \text{(A.6.9)}$$

Having found the power for both components of the input, it is straightforward to obtain the total power for an input with the signal and noise combined with a particular SNR.

$$\Phi_3(z)=\left(\frac{\sigma}{1+\sigma}\right)\frac{z\left(1-\beta^2\right)}{(z-\beta)(1-\beta z)}+\frac{1}{1+\sigma} \qquad \text{(A.6.10)}$$

(A.6.10) gives us the total input power, the denominator of the network gain. To get the total output power (i.e. the numerator of the network gain), we compute the following integral [11]:

$$\frac{1}{2\pi i}\oint_{\substack{Unit\\Circle}} TF(z)TF\left(\frac{1}{z}\right)\Phi_i(z)\frac{dz}{z} \qquad \text{(A.6.11)}$$

Rather than computing (A.6.11) for the entire input at once, we, again, compute it separately for the signal and the noise, and then take a weighted sum of the two. Therefore, for the signal:

$$I_s=\frac{1}{2\pi i}\oint_{\substack{\text{Unit}\\\text{Circle}}}\frac{z-\alpha}{z-b}\frac{\frac{1}{z}-\alpha}{\frac{1}{z}-b}\frac{z\left(1-\beta^2\right)}{(z-\beta)(1-\beta z)}\frac{dz}{z} \qquad \text{(A.6.12)}$$

Simplifying:

$$\frac{1}{2\pi i}\oint_{\substack{\text{Unit}\\\text{Circle}}}\frac{z-\alpha}{z-b}\frac{1-\alpha z}{1-bz}\frac{1-\beta^2}{(z-\beta)(1-\beta z)}dz \qquad \text{(A.6.13)}$$

To solve this, we use the residue theorem. There are 4 roots of (A.6.13):

$$\begin{aligned} z_1 &= b=\alpha(1-\Lambda)\\ z_2 &= 1/b\\ z_3 &= \beta\\ z_4 &= 1/\beta \end{aligned} \qquad \text{(A.6.14)}$$

We need to take the residue of the integrand at the roots that are within the region inscribed by the unit circle (i.e. $|z_i|<1$). For each pair of roots, z_1 and z_2 as well as z_3 and z_4, one of the pair is within the unit circle, and the other is not.

Comparing z_3 and z_4, $z_3=\beta<1$. However, comparing z_1 and z_2, we don't know, a priori, which of the two roots is less than 1. However, without loss of generality, we can assume that $z_1<1$. Therefore, we must find the residues at z_1 and z_3. This gives:

$$\frac{(b-\alpha)(1-\alpha b)}{1-b^2}\frac{1-\beta^2}{(b-\beta)(1-\beta b)}+\frac{\beta-\alpha}{\beta-b}\frac{1-\alpha\beta}{1-b\beta} \qquad \text{(A.6.15)}$$

Substituting for $b = \alpha(1 - \Lambda)$, expanding, and simplifying, we get:

$$I_s = \frac{\alpha^3\beta(\Lambda-1)+\alpha^2\beta(1-2\Lambda)+\alpha\beta(\Lambda+1)-1}{\left(\alpha^2(\Lambda-1)^2-1\right)\cdot\left(\alpha\beta(\Lambda-1)+1\right)} \qquad \text{(A.6.16)}$$

Repeating this process for the uncorrelated noise component of the input, and substituting (A.6.4) and (A.6.9) into (A.6.11), we have:

$$I_\varepsilon = \frac{1}{2\pi i}\oint_{\substack{\text{Unit}\\\text{Circle}}} \frac{z-\alpha}{z-b}\frac{1-\alpha z}{1-bz}\frac{dz}{z} \qquad \text{(A.6.17)}$$

As before, we compute the roots. This time, there are only 3:

$$\begin{aligned} z_1 &= 0 \\ z_2 &= b = \alpha(1-\Lambda) \\ z_3 &= \frac{1}{b} \end{aligned} \qquad \text{(A.6.18)}$$

Clearly, z_1 is within the unit circle. As before, without loss of generality, we pick z_2 to be the root within the unit circle. Therefore, we can use the residues at z_1 and z_2:

$$\frac{1}{1-\Lambda}-\frac{1-\alpha^2(1-\Lambda)}{1-\alpha^2(1-\Lambda)^2}\frac{\Lambda}{1-\Lambda} \qquad \text{(A.6.19)}$$

Simplifying, we have:

$$I_\varepsilon = \frac{\alpha^2(1-2\Lambda)-1}{\alpha^2(\Lambda-1)^2-1} \qquad \text{(A.6.20)}$$

We can compute the total output power for an input mixture of signal and noise, with SNR, σ, in the following way:

$$I = \frac{\sigma}{1+\sigma}I_s + \frac{1}{1+\sigma}I_\varepsilon \qquad \text{(A.6.21)}$$

Substituting, we have:

$$I=\left(\frac{\sigma}{1+\sigma}\right)\cdot\frac{\alpha^{3}\beta(\Lambda-1)+\alpha^{2}\beta(1-2\Lambda)+\alpha\beta(\Lambda+1)-1}{\left(\alpha^{2}(\Lambda-1)^{2}-1\right)\cdot\left(\alpha\beta(\Lambda-1)+1\right)}+\left(\frac{1}{1+\sigma}\right)\frac{\alpha^{2}(1-2\Lambda)-1}{\alpha^{2}(\Lambda-1)^{2}-1} \quad \text{(A.6.22)}$$

We must now find the parameters, α and $\Lambda\cdot\Lambda$, that minimize (A.6.22). The straightforward way to do so would be to take the two derivatives of (A.6.22) with respect to each of α and Γ and then set the derivatives to 0. However, it has not been possible to solve these derivatives. Instead, examining the form of the derivatives, we see that the following expression is much more compact.

$$\frac{\partial I}{\partial\alpha}-\frac{\partial I}{\partial\Gamma}\frac{\Lambda-1}{\alpha}$$

Now, if $\frac{\partial I}{\partial\alpha}=\frac{\partial I}{\partial\Lambda}=0$, then:

$$\frac{\partial I}{\partial\alpha}-\frac{\partial I}{\partial\Lambda}\frac{\Lambda-1}{\alpha}=0 \quad \text{(A.6.23)}$$

Therefore, if we can solve (A.6.23) for either variable, and then substitute back into the other derivative, we can find the optimum for both variables. Computing (A.6.23) and then simplifying gives us:

$$-\frac{2\left(\alpha\cdot\Lambda\cdot(1+\beta\cdot\sigma)+\alpha^{2}\beta(\Lambda-1)(\Lambda-\sigma)-\beta\cdot\sigma\right)}{\left(-1+\alpha^{2}(\Lambda-1)^{2}\right)\left(1+\alpha\cdot\beta(\Lambda-1)\right)(1+\sigma)}=0 \quad \text{(A.6.24)}$$

(A.6.24) is quadratic in each of the two variables of interest: α and Γ. Therefore, solving for each of these variables in (A.6.24) gives us a solution from the quadratic equation:

$$\alpha=\frac{-\Lambda\cdot(1+\beta\cdot\sigma)\pm\sqrt{\Lambda^{2}(1+\beta\cdot\sigma)^{2}+4\sigma\cdot\beta^{2}(\Lambda-1)(\Lambda-\sigma)}}{2\beta(\Lambda-1)(\Lambda-\sigma)} \quad \text{(A.6.25)}$$

$$\Gamma=\frac{(\alpha\beta+\alpha\beta\sigma-\beta\sigma-1)\pm\sqrt{\alpha^{2}\beta^{2}(\sigma-1)^{2}-2a\beta(1+\sigma)(1+\beta\sigma)+\beta^{2}\sigma(1+\sigma)+2\beta\sigma+1}}{2\alpha\cdot\beta} \quad \text{(A.6.26)}$$

Let $g=(\Lambda-1)(\Lambda-\sigma)$. Then, substituting into (A.6.25) and simplifying, we get:

$$\alpha = \beta \left\{ -\frac{\Lambda \cdot (1+\beta\sigma)}{2\beta^2 g} \pm \frac{\sqrt{\Lambda^2 (1+\beta\sigma)^2 + 4\sigma\beta^2 g}}{2\beta^2 g} \right\} \qquad \text{(A.6.27)}$$

Let us assume that the term within the curly brackets in (A.6.27) is equal to 1. Given this assumption, $\alpha = \beta$. Further, if we simplify the term in the curly brackets, we get:

$$\pm\sqrt{\Lambda^2 (1+\beta\sigma)^2 + 4\beta^2 g} = 2\beta^2 g + \Lambda \cdot (1+\beta\sigma)$$

$$\beta^2 g + \Lambda \cdot (1+\beta\sigma) - 1 = 0 \qquad \text{(A.6.28)}$$

Substituting back for g, we can now solve (A.6.28) for Λ. Therefore,

$$\Lambda = \frac{(\beta^2\sigma + \beta^2 - \beta\sigma - 1) \pm \sqrt{(\beta^2\sigma + \beta^2 - \beta\sigma - 1)^2 + 4\beta^2\sigma(1-\beta^2)}}{2\beta^2} \qquad \text{(A.6.29)}$$

As explained earlier, the assumption that we made forces $\alpha = \beta$. If we now substitute that into (A.6.26), we get the following equation:

$$\Lambda = \frac{(\beta^2\sigma + \beta^2 - \beta\sigma - 1) \pm \sqrt{\beta^4 (\sigma - 1)^2 - 2\beta^2 (1+\sigma)(1+\beta\sigma) + \beta^2\sigma(1+\sigma) + 2\beta\sigma + 1}}{2\beta \cdot \beta} \text{(A.6.30)}$$

By inspection, (A.6.30) and (A.6.29) are exactly the same. Therefore, we have found, by inspection, a solution of (A.6.23).

This solution takes the following form (where we add the * to denote the optimality of the parameters):

$$e^{-1/\tau} = \alpha^* = \beta = e^{-1/\tau_s} \qquad \text{(A.6.31)}$$

$$\Lambda^* = \frac{(\beta^2\sigma + \beta^2 - \beta\sigma - 1) + \sqrt{(\beta^2\sigma + \beta^2 - \beta\sigma - 1)^2 + 4\beta^2\sigma(1-\beta^2)}}{2\beta^2} \qquad \text{(A.6.32)}$$

(A.6.31) and (A.6.32) directly provide the form of the optimal parameters used in the text.

Expanding the feedforward recursion

Starting from Eq 9:

$$p_t = f_t - n_t \quad \text{and} \quad n_t = \hat{\alpha}\left(n_{t-1} + \hat{\Gamma} f_{t-1}\right) \qquad \text{(A.7.1)}$$

In the feedback circuit, the interneuron does not depend on the output of the principal neuron. Hence, the two equations can be solved independently.

$$n_t = \hat{\alpha} \cdot n_{t-1} + \hat{\alpha} \cdot \hat{\Gamma} f_{t-1} \qquad \text{(A.7.2)}$$

Therefore, substituting for n_{t-1}, we get:

$$n_t = \hat{\alpha} \cdot \left(\hat{\alpha} \cdot n_{t-2} + \hat{\alpha} \cdot \hat{\Gamma} f_{t-2} \right) + \hat{\alpha} \cdot \hat{\Gamma} f_{t-1} = \hat{\alpha} \cdot \hat{\Gamma} f_{t-1} + \hat{\alpha}^2 \cdot \hat{\Gamma} f_{t-2} + \hat{\alpha}^2 \cdot n_{t-2} \qquad \text{(A.7.3)}$$

Continuing this recursive substitution, we have:

$$n_t = \hat{\Gamma} \cdot \sum_{i=1}^{\infty} \hat{\alpha}^i \cdot f_{t-i} \qquad \text{(A.7.4)}$$

Substituting back into the function for the principal neuron:

$$p_t = f_t - \hat{\Gamma} \cdot \sum_{i=1}^{\infty} \hat{\alpha}^i \cdot f_{t-i} \qquad \text{(A.7.5)}$$

as shown in the text in Eq 10.

Stability of Neural Implementations of Predictive Coding

A network is potentially unstable to disturbances if a disturbance can loop through the circuit and get amplified across each loop. Therefore, a purely feedforward network, as in Figure 2a, is stable because it does not contain any loops. In contrast, a feedback network can be unstable if the gain across the loop is greater than 1. However, in our feedback circuit, the only non-unitary gain is the feedback gain, Γ. As detailed above, since $\Gamma = \Lambda^*$, Γ is always less than 1 (Figure 1b) (independent of the precise value for β). Therefore, the gain of the loop in the feedback network implementing predictive coding is always less than 1, and the feedback circuit is stable to any internal disturbances; noise within the circuit will not amplify across the loop. Similarly, given the fact that the nonlinearity in the nonlinear feedback network acts to change the gain of the feedback neuron, but that the gain is still never increased beyond 1, the nonlinear network is also stable to internal disturbances. Therefore, this analysis shows that the linear feedforward, linear feedback, and nonlinear feedback implementations of predictive coding are all stable with respect to noise within the circuit.

Linear filter shift is due to feedback inhibitory structure

The use of the modified, three neuron, three time-constant model in simulating responses for comparing with experiment might raise the concern that the agreement with the experiment results is due to the additional parameters. We show, using an analytical analyses of the first trough in the linear filter, that the qualitative shift in this part of the filter can be observed, even in simpler models (with fewer parameters), and is not dependent on the specific parameter values.

It is not possible to analytically solve the response of the nonlinear circuit. Therefore, we have to find a way of approximating its responses, with a linear model. Transmitting a signal of two different amplitudes through a rectilinear nonlinearity effectively results in a change in the effective gain of the response (if one attempts to linearize the transfer, for each input, separately). To demonstrate this, consider a rectilinear function:

$$R_\delta(x) = \begin{cases} x+\delta & x<-\delta \\ 0 & -\delta<x<\delta \\ x-\delta & \delta<s \end{cases} \qquad \text{(A.9.1)}$$

Let us choose δ = 1 and plot the resulting response:

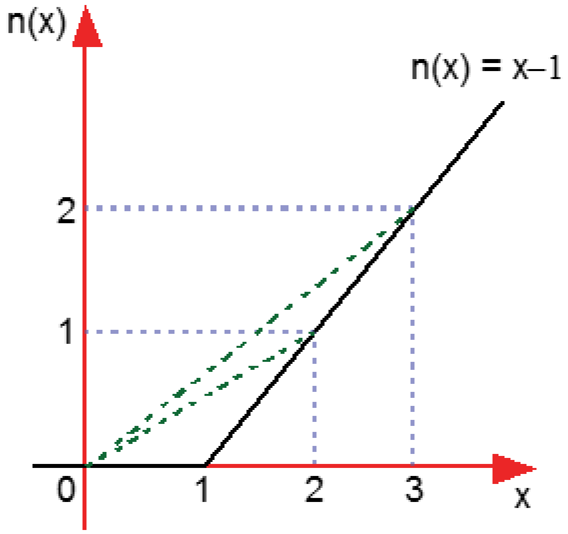

Therefore, one way of approximating the nonlinear model would be to consider the linear transfer function, for a model, where the gain of the feedback circuit was changed. Increasing the amplitude of the input in a nonlinear model would be equivalent to an increased gain, and vice versa for a decrease in the amplitude. We will use the method in the analysis, below.

Given this, we still have to identify a linear model that would show the change in the location of the first extremum of the filter. If we simply utilize a two cell model, where the projection neuron has a time constant of 0, then we can describe the linear filter as a delayed exponential filter subtracted from a delta function. Therefore, the extremum (defined by the subtracted exponential filter) cannot shift in time.

Therefore, we must introduce at least a second time constant. Specifically, we utilize the model diagrammed in Fig S4b, with an inputcell   to the nonlinear predictive coding circuit, which has a non-zero time constant. Since, in most real circuits, the predictive coding element would be embedded within a larger circuit (with some non-zero temporal response pattern), this is entirely reasonable.

Now, we know that the analytical form of the expression for the original two cell model. In a slightly modified form from (), it is:

$$p_t = f_t - \Gamma \cdot \sum_{i=1}^{\infty} \alpha^i \left(1-\Gamma\right)^{i-1} f_{t-i} \qquad \text{(A.9.2)}$$

From the solution of a single neuron, with a given time constant, we know the analytical form for the input to the two cell model, from the third cell:

$$f_t = \sum_{k=0}^{\infty} \chi^k g_{t-k} \tag{A.9.3}$$

Substituting (A.9.3) into (A.9.2), and performing a change of variables in the second term, we have:

$$p_t = \sum_{j=0}^{\infty} \chi^j f_{t-j} - \Gamma \cdot \sum_{i=1}^{\infty} \left(\alpha^i (1-\Gamma)^{i-1} \left[\sum_{j=i}^{\infty} \gamma^{j-i} f_{t-j} \right] \right) \tag{A.9.4}$$

Inverting the sum in the second term (using geometric intuition), we get:

$$\sum_{j=1}^{\infty} \left(\left[\sum_{i=1}^{j} \left(\alpha^i (1-\Gamma)^{i-1} \chi^{-i} \right) \right] \chi^j f_{t-j} \right) \tag{A.9.5}$$

The term within the square brackets is a geometric series, and can be computed analytically. (Indeed, even convergence is not necessary, because the series is bounded.)

$$\sum_{i=1}^{j} \left(\alpha^i (1-\Gamma)^{i-1} \chi^{-i} \right) = \frac{\alpha}{\chi^j} \left[\frac{\chi^j - (\alpha(1-\Gamma))^j}{\chi - \alpha(1-\Gamma)} \right] \tag{A.9.6}$$

Substituting (A.9.6) and (A.9.5) back into (A.9.4), we get:

$$\sum_{j=0}^{\infty} \chi^j f_{t-j} - \Gamma \alpha \sum_{j=1}^{\infty} \left(\left[\frac{\chi^j - (\alpha(1-\Gamma))^j}{\chi - \alpha(1-\Gamma)} \right] f_{t-j} \right) \tag{A.9.7}$$

From (A.9.7), we can read off the form of the weighting filter, for each value of α, γ, and Γ, at a given time point, $t = j$.

$$L_j = \chi^j - \alpha \cdot \Gamma \cdot \left[\frac{\chi^j - (\alpha(1-\Gamma))^j}{\chi - \alpha(1-\Gamma)} \right] \tag{A.9.8}$$

We would now like to study the shape of this function. First, we note that $L_0 = 1$. Second, as $j \to \infty$, $L_j \to 0$. (This is because $0 < \alpha, \chi, \Gamma < 1$.) Knowing these two limits tells us that, if L_j has a zero for some $j > 0$, then there must be at least one local minimum in the linear filter. Therefore, we solve for j_0 such that $L_j = 0$.

$$j_0 = \frac{\ln\left(\frac{\alpha \cdot \Gamma}{\alpha - \chi} \right)}{\ln\left(\frac{\chi}{\alpha(1-\Gamma)} \right)} \tag{A.9.9}$$

In solving to obtain this root, we made the inherent assumption that the terms within the logarithms are both greater than 0. For the term in the denominator, this is always true. However, for the term in the numerator, this means that $\alpha > \chi$. This is reasonable, since it only necessitates that the interneuron should have a longer time constant than the neuron on the feedforward path.

Changing a variable in (A.9.9) allows us to reduce one free parameter.

$$j_0 = \frac{\ln\left(\frac{\Gamma}{\gamma}\right)}{\ln\left(\frac{1-\gamma}{1-\Gamma}\right)} \qquad \text{(A.9.10)}$$

where $\chi = \alpha(1-\gamma)$. Since both $0 < \alpha, \chi < 1$,

$$0 < \alpha - \chi = \alpha\gamma < 1$$

And therefore,

$$0 < \gamma < 1 \qquad \text{(A.9.11)}$$

We wish to show that $j_0 > 0$, for all allowed values of the parameters. To simplify this, we divide the space of parameters into two regimes. First, we fix a value of $\gamma = \bar{\gamma}$, and then consider the regime where $\Gamma > \bar{\gamma}$. In this case, $1-\Gamma < 1-\bar{\gamma}$. Therefore,

$$\frac{\Gamma}{\bar{\gamma}} > 1 \qquad \text{(A.9.12)}$$

and

$$\frac{1-\bar{\gamma}}{1-\Gamma} > 1 \qquad \text{(A.9.13)}$$

Notice that the natural logarithm changes sign when its input is equal to 1. Therefore, given that both the numerator and the denominator of (A.9.10) act on terms that are > 1, the signs of both the numerator and denominator are positive, and $j_0 > 0$.

Now, consider the second regime, where $\Gamma < \bar{\gamma}$. Then, $1-\Gamma > 1-\bar{\gamma}$. Then,

$$\frac{\Gamma}{\bar{\gamma}} < 1 \qquad \text{(A.9.14)}$$

$$\frac{1-\bar{\gamma}}{1-\Gamma} < 1 \qquad \text{(A.9.15)}$$

Again, both the logarithms in the numerator and denominator act on terms that are < 1. This means that both numerator and denominator will be negative, and, therefore, $j_0 > 0$.

Therefore, making only the assumption that $\alpha > \chi$, the linear filter for the modified predictive coding network has a crossing point and, therefore, a local minimum within the response function.

To show that the linear filter shifts, closer to t = 0, as the gain increases (so as to model the effect of increasing the amplitude of the input in a nonlinear network), we study the location of the cross-over point, as Γ varies. We could also study the location of the local minimum, but the result is analogous, and, for simplicity, we go over only one here.

Therefore, we consider how (A.9.10) varies, as Γ varies, for a fixed $\bar{\gamma}$.

$$j_0 = \frac{\ln\left(\frac{\Gamma}{\bar{\gamma}}\right)}{\ln\left(\frac{1-\bar{\gamma}}{1-\Gamma}\right)}$$

Consider, first, a very small Γ (i.e. small with respect to $\bar{\gamma}$). Then:

$$j_0 \approx \frac{\ln(\Gamma)}{C} \qquad \text{(A.9.16)}$$

where C is a fixed constant (which depends only on $\bar{\gamma}$). Therefore, as $\Gamma \to 0$, for any particular fixed $\bar{\gamma}$, $j_0 \to \infty$.

In contrast, we can consider $\Gamma \to 1$. Then:

$$j_0 \approx \frac{C'}{\ln\left(\frac{1}{1-\Gamma}\right)} \qquad \text{(A.9.17)}$$

Again, C' is a fixed constant (that depends only on $\bar{\gamma}$). Clearly, the denominator approaches ∞. Therefore, as $\Gamma \to 1, j_0 \to 0$.

These two limiting cases, suggest that, as the feedback strength increases, the location of the cross-over point in the linear filter reduces towards 0. To confirm this, we need to show that the function is monotonic decreasing.

Rewriting (A.9.10), we have:

$$j_0 = \frac{\ln(\Gamma)-\ln(\bar{\gamma})}{\ln(1-\bar{\gamma})-\ln(1-\Gamma)} = \frac{\ln(\Gamma)-D}{D'-\ln(1-\Gamma)} \qquad \text{(A.9.18)}$$

where D, D' are arbitrary constants, dependent only on $\bar{\chi}$. Note that, because $0<\bar{\gamma}<1$, we know that both $D, D' < 0$.

Both the numerator and denominator of (A.9.18) are monotonic functions. As Γ increases, $\ln(\Gamma)$ monotonically decreases the absolute value of its amplitude (approaching 0). In contrast, as Γ increases, $\ln(1-\Gamma)$ monotonically increases the absolute value of its amplitude (approaching ∞).

Therefore, the numerator of (A.9.18) increases from $-\infty$ to $|D|$. In contrast, the denominator varies from D' to ∞. Because we know the sign of the ratio (i.e. j_0) never changes, the two functions must approach zero (and cross over from negative to positive) at exactly the same point – when $\Gamma = \bar{\gamma}$. Since the limit of both the numerator and denominator, at this point, is 0, the discontinuity at this point is removable. Therefore, the function for j_0 does not diverge around $\Gamma = \bar{\gamma}$, but is simply undefined. Therefore, we can conclude that (A.9.18) is a monotonic function. Further, because of the limiting values, we see that it is monotonic decreasing, as Γ increases.

As input to a nonlinear feedback inhibitory network increases in amplitude, the effective linear gain of the feedback circuit is increased. Therefore, we have been able to show, analytically, with the use of only a single assumption on the parameter space (and the addition of only a single parameter, χ), that the nonlinear predictive coding network will result in a shift in the linear filter towards $t = 0$, as the amplitude of the input increase. This is exactly the result that we wanted to confirm, and confirms that the shift in the linear response filter of the nonlinear predictive coding network is a qualitative property of the underlying feedback inhibitory network, and not the specific parameter values [45].
